# Single low-dose tafenoquine combined with dihydroartemisinin–piperaquine to reduce *Plasmodium falciparum* transmission in Ouelessebougou, Mali: a phase 2, single-blind, randomised clinical trial

**DOI:** 10.1016/S2666-5247(21)00356-6

**Published:** 2022-05

**Authors:** Will Stone, Almahamoudou Mahamar, Merel J Smit, Koualy Sanogo, Youssouf Sinaba, Sidi M Niambele, Adama Sacko, Sekouba Keita, Oumar M Dicko, Makonon Diallo, Seydina O Maguiraga, Siaka Samake, Oumar Attaher, Kjerstin Lanke, Rob ter Heine, John Bradley, Matthew B B McCall, Djibrilla Issiaka, Sekou F Traore, Teun Bousema, Chris Drakeley, Alassane Dicko

**Affiliations:** a Department of Infection Biology, London School of Hygiene & Tropical Medicine, London, UK; b MRC International Statistics and Epidemiology Group, London School of Hygiene & Tropical Medicine, London, UK; c Malaria Research and Training Centre, Faculty of Pharmacy and Faculty of Medicine and Dentistry, University of Sciences Techniques and Technologies of Bamako, Bamako, Mali; d Department of Medical Microbiology and Radboud Center for Infectious Diseases, Radboud University Medical Center, University of Nijmegen, Nijmegen, Netherlands; e Department of Pharmacy and Radboud Center for Infectious Diseases, Radboud University Medical Center, University of Nijmegen, Nijmegen, Netherlands

## Abstract

**Background:**

Tafenoquine was recently approved as a prophylaxis and radical cure for *Plasmodium vivax* infection, but its *Plasmodium falciparum* transmission-blocking efficacy is unclear. We aimed to establish the efficacy and safety of three single low doses of tafenoquine in combination with dihydroartemisinin–piperaquine for reducing gametocyte density and transmission to mosquitoes.

**Methods:**

In this four-arm, single-blind, phase 2, randomised controlled trial, participants were recruited at the Clinical Research Unit of the Malaria Research and Training Centre of the University of Bamako in Mali. Eligible participants were aged 12–50 years, with asymptomatic *P falciparum* microscopy-detected gametocyte carriage, had a bodyweight of 80 kg or less, and had no clinical signs of malaria defined by fever. Participants were randomly assigned (1:1:1:1) to standard treatment with dihydroartemisinin–piperaquine, or dihydroartemisinin–piperaquine plus a single dose of tafenoquine (in solution) at a final dosage of 0·42 mg/kg, 0·83 mg/kg, or 1·66 mg/kg. Randomisation was done with a computer-generated randomisation list and concealed with sealed, opaque envelopes. Dihydroartemisinin–piperaquine was administered as oral tablets over 3 days (day 0, 1, and 2), as per manufacturer instructions. A single dose of tafenoquine was administered as oral solution on day 0 in parallel with the first dose of dihydroartemisinin–piperaquine. Tafenoquine dosing was based on bodyweight to standardise efficacy and risk variance. The primary endpoint, assessed in the per-protocol population, was median percentage change in mosquito infection rate 7 days after treatment compared with baseline. Safety endpoints included frequency and incidence of adverse events. The final follow-up visit was on Dec 23, 2021; the trial is registered with ClinicalTrials.gov, NCT04609098.

**Findings:**

From Oct 29 to Nov 25, 2020, 1091 individuals were screened for eligibility, 80 of whom were enrolled and randomly assigned (20 per treatment group). Before treatment, 53 (66%) individuals were infectious to mosquitoes, infecting median 12·50% of mosquitoes (IQR 3·64–35·00). Within-group reduction in mosquito infection rate on day 7 was 79·95% (IQR 57·15–100; p=0·0005 for difference from baseline) following dihydroartemisinin–piperaquine only, 100% (98·36–100; p=0·0005) following dihydroartemisinin–piperaquine plus tafenoquine 0·42 mg/kg, 100% (100–100; p=0·0001) following dihydroartemisinin–piperaquine plus tafenoquine 0·83 mg/kg, and 100% (100–100; p=0·0001) following dihydroartemisinin–piperaquine plus tafenoquine 1·66 mg/kg. 55 (69%) of 80 participants had a total of 94 adverse events over the course of the trial; 86 (92%) adverse events were categorised as mild, seven (7%) as moderate, and one (1%) as severe. The most common treatment-related adverse event was mild or moderate headache, which occurred in 15 (19%) participants (dihydroartemisinin–piperaquine n=2; dihydroartemisinin–piperaquine plus tafenoquine 0·42 mg/kg n=6; dihydroartemisinin–piperaquine plus tafenoquine 0·83 mg/kg n=3; and dihydroartemisinin–piperaquine plus tafenoquine 1·66 mg/kg n=4). No serious adverse events occurred. No significant differences in the incidence of all adverse events (p=0·73) or treatment-related adverse events (p=0·62) were observed between treatment groups.

**Interpretation:**

Tafenoquine was well tolerated at all doses and accelerated *P falciparum* gametocyte clearance. All tafenoquine doses showed improved transmission reduction at day 7 compared with dihydroartemisinin–piperaquine alone. These data support the case for further research on tafenoquine as a transmission-blocking supplement to standard antimalarials.

**Funding:**

Bill & Melinda Gates Foundation.

**Translations:**

For the French, Portuguese, Spanish and Swahili translations of the abstract see Supplementary Materials section.

**Research in context**

**Evidence before this study**

Primaquine and its long-acting analogue, tafenoquine, belong to the class of 8-aminoquinolines that can clear *Plasmodium vivax* liver stages and thereby form an essential component of *P vivax* radical cure. Primaquine is also a potent *Plasmodium falciparum* gametocytocide; a single low dose of primaquine combined with standard artemisinin-combination therapy (ACT) prevents transmission by rapidly sterilising and killing *P falciparum* gametocytes. The potency of tafenoquine as a *P falciparum* gametocytocide has so far remained unstudied. We searched PubMed on June 22, 2021, with no restrictions on publication date or language, for studies assessing the transmission-blocking and gametocytocidal abilities of tafenoquine treatment with search terms: ([Tafenoquine] OR [Krintafel] OR [Arakoda] OR [WR-238605]) AND ([Gametocytocidal] OR [Gametocytes] OR [Transmission]). Among the 16 results (1992–2019) meeting the search criteria, none of the identified studies assessed the *P falciparum* gametocytocidal and transmission-blocking properties of tafenoquine in naturally infected gametocyte carriers. The results consisted of the following: four reviews; two studies assessing the sporontocidal activity of tafenoquine against *P vivax*; one study assessing glucose-6-phosphate dehydrogenase deficiency prevalence in Ethiopia; one study comparing in-vitro sensitivity of *P falciparum* isolates against three 8-aminoquinolines; one study investigating drug interactions between tafenoquine and ACTs; two articles regarding the development of tafenoquine; one study in which the prophylactic efficacy of tafenoquine was studied in a human *P falciparum* challenge model; and four studies wherein gametocytocidal and sporontocidal activity of tafenoquine was tested in avian and rodent models.

**Added value of this study**

Transmission-blocking antimalarials are important components of strategies to eliminate malaria and prevent the spread of drug-resistant *Plasmodium*. The long-acting 8-aminoquinoline tafenoquine might have unique properties in blocking the transmission of malaria over a prolonged period and possibly multiple infections. To our knowledge, this is the first clinical trial assessing the *P falciparum* gametocytocidal and transmission-blocking properties of single low doses of tafenoquine. Three different single low doses of tafenoquine were combined with dihydroartemisinin–piperaquine. The recommended dose of tafenoquine for radical cure of *P vivax* is 300 mg (ie, 5 mg/kg in an adult weighing 60 kg). The maximum dose used in the current study was one third of this recommended dose (1·66 mg/kg, equivalent to 100 mg total dose in an adult weighing 60 kg). The results show that tafenoquine accelerated *P falciparum* gametocyte clearance in a dose-dependent manner and greatly reduced transmission. Although findings from previous studies suggest that primaquine's efficacy is evident within the first 48 h after administration, tafenoquine's transmission-blocking occurred later; first detected on day 7 after initiation of treatment. These data support the case for further research on tafenoquine as a transmission-blocking supplement to standard antimalarials.

**Implications of all the available evidence**

This study provides the first evidence that tafenoquine is gametocytocidal in humans and prevents transmission to mosquitoes by day 7 after treatment. Some individuals treated with dihydroartemisinin–piperaquine alone were still infectious to mosquitoes up to the last day of mosquito feeding assays (day 14 after treatment), corroborating earlier reports on the persistence of transmissible gametocytes after artemisinin-combination therapy. By contrast, none of the individuals who received tafenoquine were infectious by day 14 and the majority of transmission events were prevented by day 7. The current findings highlight the importance of gametocytocidal and transmission-blocking supplements in areas trying to reduce the malaria infectious reservoir and identify tafenoquine as a new *P falciparum* gametocytocide.

## Introduction

After years of success in malaria control, efforts to further reduce the global incidence of malaria are stalled. There were an estimated 229 million cases of malaria worldwide in 2019, up from 217 million in 2014.^1^ With spreading resistance to first-line drugs against *Plasmodium falciparum* malaria, there is a clear need for interventions focused on reducing malaria parasite transmission.

Artemisinin combination therapies (ACTs) clear *P falciparum* asexual parasites but have little activity against mature gametocytes. Gametocytes are the only *Plasmodium* life stage that can be transmitted to mosquitoes, so their prevalence, density, and distribution within the human population define the malaria infectious reservoir. In areas consolidating malaria elimination or aiming to contain the spread of artemisinin resistance, WHO recommends that ACTs be combined with a single low dose of the 8-aminoquinoline compound, primaquine.^2^ Single low-dose primaquine combined with ACT prevents transmission by rapidly sterilising and killing gametocytes, but its short half-life (4–9 h) might prevent primaquine from affecting any gametocytes that arise late during infections or develop after recrudescence. The effect of single low-dose primaquine might therefore be limited to the sterilisation of currently circulating gametocytes.^3^ Another 8-aminoquinoline, tafenoquine, has recently been approved as a single-dose radical cure for *Plasmodium vivax*^4^ and its potential as a *P falciparum* gametocytocide is suggested by in-vitro^5^ and murine models.^6^ The long half-life (15 days)^7^ of tafenoquine could be a major advantage over short-lived gametocytocidal treatments, but comes with a risk of prolonged haemolysis for individuals deficient in the production of glucose-6-phosphate dehydrogenase (G6PD),^8^ who can have acute haemolytic anaemia after radical curative treatment of *P vivax* malaria with primaquine. Recent trials indicate that a single dose of 300 mg tafenoquine is considered safe among individuals with normal G6PD production and has equivalent transient haemolytic activity to standard 14-day primaquine regimens for *P vivax* treatment in women who are deficient in G6PD.^8^

Tafenoquine has not yet been tested in controlled trials for its ability to clear and prevent transmission of *P falciparum* gametocytes. Dosing for a gametocytocidal indication has not been assessed but is likely to be substantially lower than the required dose for radical cure of *P vivax* (as is the case with primaquine). In the current study, we assessed the gametocytocidal and transmission-blocking efficacy of a range of single low doses of tafenoquine (0·41 mg/kg, 0·83 mg/kg, and 1·66 mg/kg) in combination with dihydroartemisinin–piperaquine in Malian children and adults with normal G6PD production.

## Methods

### Study design and participants

This four-arm, single-blind, phase 2, randomised controlled trial was conducted at the Clinical Research Unit of the Malaria Research and Training Centre (MRTC) of the University of Bamako in Mali. Before the commencement of screening, our study team of clinicians and technicians met with community leaders, village health workers, and heads of households from each village to explain the study and obtain approval to conduct the study. Village health workers then used a door-to-door approach to inform households of the date and location where consenting and screening would take place. Participants were included in the trial if they met the following criteria: positive for *P falciparum* gametocytes by microscopy (ie, ≥1 gametocytes recorded in a thick film against 500 white blood cells, equating to 16 gametocytes per μL with a standard conversion of 8000 white blood cells per μL blood); absence of other non-*P falciparum* species on blood film; haemoglobin density of 10 g/dL or more; normal G6PD (male >4 IU/g haemoglobin, female >6 IU/g haemoglobin); aged between 12–50 years; bodyweight 80 kg or less; no clinical signs of malaria defined by fever (≥37·5°C); no signs of acute, severe, or chronic disease; no allergies to any study drugs; reported no use of antimalarial drugs over the past week; consistent with the long half-life of tafenoquine, use of effective contraception for five half-lives (3 months) after the end of tafenoquine treatment. Exclusion criteria included pregnancy (tested at enrolment by urine and serum test) or lactation, use of other medication (except for paracetamol or aspirin), family history of congenital prolongation of the corrected QT interval, current or recent treatment with drugs known to extend the corrected QT interval, and blood transfusion in the past 90 days. Before screening and before study enrolment, participants provided written informed consent (aged ≥18 years) or assent with written parental consent (aged 12–17 years).

Ethical approval was granted by the Ethics Committee of the Faculty of Medicine, Pharmacy, and Dentistry of the University of Science, Techniques, and Technologies of Bamako (Bamako, Mali), and the Research Ethics Committee of the London School of Hygiene & Tropical Medicine (London, UK).

### Randomisation and masking

Participants were randomly assigned (1:1:1:1) to receive dihydroartemisinin–piperaquine plus tafenoquine 1·66 mg/kg, dihydroartemisinin–piperaquine plus tafenoquine 0·83 mg/kg, dihydroartemisinin–piperaquine plus tafenoquine 0·42 mg/kg, or dihydroartemisinin–piperaquine alone. Enrolment continued until 80 participants were enrolled (20 individuals assigned to each treatment group). An independent MRTC statistician randomly generated the treatment assignment using Stata (version 16), which was linked to participant identification number. The statistician prepared sealed, opaque envelopes with the participant identification number on the outside and treatment assignment inside, which were sent to the MRTC study pharmacist. The study pharmacist provided treatment according to the contained assignment and was consequently not masked to treatment assignment. All other investigators and staff involved in assessing all laboratory outcomes were masked. Participants could ask the study physician which treatment they received.

### Procedures

Dihydroartemisinin–piperaquine was administered as oral tablets over 3 days (day 0, 1, and 2), as per manufacturer instructions (see appendix 5 p 2 for dosing). A single dose of tafenoquine was administered as oral solution on day 0 in parallel with the first dose of dihydroartemisinin–piperaquine. Tafenoquine dosing was based on bodyweight to standardise efficacy and risk variance (appendix 5 pp 2–3). The maximum dose chosen for the current study was based on equivalent safety profile of tafenoquine doses of 300 mg or less and to standard primaquine dosing (15 mg daily for 14 days) in G6PD heterozygous adults (full details in appendix 5 pp 2–3).^8^ The recommended dose of tafenoquine for radical cure of *P vivax* is 300 mg (ie, 5 mg/kg in an adult weighing 60 kg).^7^ The maximum dose used in the current study was 1·66 mg/kg, equivalent to 100 mg total dose in an adult weighing 60 kg. G6PD testing was done using both semiquantitative (OSMMR-D G-6-PD Test; R&D Diagnostics, Aghia Paraskevi, Greece) and quantitative testing (STANDARD G6PD Test; SD Biosensor, Suwon, South Korea); inclusion in the study required normal enzyme function to be determined by both methods. The thresholds used for normal G6PD activity were more than 30% for male participants and 70% for female participants.

Participants received a full clinical and parasitological examination on days 1, 2, 7, 14, 21, and 28 after receiving the first dose of the study drugs. Giemsa stained thick film microscopy was performed as described previously, with asexual stages counted against 200 white blood cells and gametocytes counted against 500 white blood cells.^9^ For molecular gametocyte quantification, total nucleic acids were extracted using a MagNAPure LC automated extractor (Total Nucleic Acid Isolation Kit-High Performance; Roche Applied Science, Indianapolis, IN, USA). Male and female gametocytes were quantified in a multiplex reverse transcriptase quantitative PCR (RT-qPCR) assay,^10^ targeting CCP4/PfMGET mRNA (appendix 5 p 5). Samples were classified as negative for a particular gametocyte sex if the RT-qPCR quantified density of gametocytes of that sex was less than 0·01 gametocytes per μL (ie, one gametocyte per 100 μL of blood sample). If accurate gametocyte quantification was not possible (eg, due to uncertainty over starting blood volume), density values were removed from analysis while prevalence values were retained. Haemoglobin density was measured using a haemoglobin analyser (HemoCue; AB Leo Diagnostics, Helsingborg, Sweden) or automatic haematology analyser (HumaCount 5D; Human Diagnostics Worldwide, Wiesbaden, Germany). Methaemoglobin was measured non-invasively using a Masimo Rainbow SET platform (Masimo Corporation, Irvine, CA, USA). Methaemoglobin saturation measures of 0% were confirmed as methodological errors and removed from analysis. Additional venous blood samples were taken for biochemical and infectivity assessments on days 0, 2, 7, and 14 in all treatment groups. Aspartate aminotransferase, alanine aminotransferase, and blood creatine concentrations were measured using automatic biochemistry analyser HumaStar 100 (Human Diagnostics Worldwide, Wiesbaden, Germany). For each assessment of infectivity, approximately 75 locally reared *Anopheles gambiae* were allowed to feed for 15–20 min on venous blood samples (Lithium Heparin VACUETTE tube; Greiner Bio-One, Kremsmünster, Austria) through a prewarmed glass membrane feeder system (Coelen Glastechniek, Weldaad, Netherlands). All surviving mosquitoes were dissected on the seventh day after the feeding assay; midguts were stained in 1% mercurochrome and examined for the presence and density of oocysts by expert microscopists.

### Outcomes

The primary outcome measure was median percentage change in mosquito infection rate between pretreatment and 7 days after treatment. Secondary outcomes were mosquito infection metrics (oocyst density, mosquito infection rate, and infectious individuals) at other prespecified timepoints (day 0, 2, 7, and 14); gametocyte prevalence, density, circulation time, area under the curve (AUC) of density over time, and sex ratio (ie, proportion of gametocytes that were male); and safety assessments including incidence of clinical and laboratory adverse events. Differences in all transmission, gametocyte, and safety outcomes were compared between treatment groups (individual tafenoquine groups compared with the reference dihydroartemisinin–piperaquine-only treatment group) as secondary outcomes.

Adverse events were graded by the study clinician for severity (mild, moderate, or severe) and relatedness to study medication (unrelated or unlikely, possibly, probably, or definitely related). A reduction in haemoglobin concentration of 40% or more from baseline was categorised as a haematological adverse event. An external data safety and monitoring committee was assembled before the trial, and safety data were discussed after enrolment of 40 participants, and after the final follow-up visit of the last participant.

### Statistical analysis

Sample size estimation was based on efficacy for single-dose primaquine (in the absence of infectivity level data on tafenoquine, and assuming equivalence) to provide a 95% or greater reduction in infectivity at 7 days after initiation of treatment compared with pretreatment using a membrane feeding assay.^11^ With 20 participants per group, we would have 80% power to detect a 95% or greater reduction in the number of mosquitoes with oocysts after treatment as significant at the 0·05 level. The sample size was designed to assess change in infectivity within treatment groups, not compare transmission reducing effects between treatment groups.

All outcomes were analysed in the per-protocol population. Mosquito infectivity was assessed at three levels: mean number of oocysts in a sample of mosquitoes (ie, oocyst density), the proportion of mosquitoes infected with any number of oocysts (ie, mosquito infection rate), and infectivity of the study participant to any number of mosquitoes (ie, infectious individuals). Asexual parasite density was not measured by molecular methods due to the scarcity of nucleic acid extraction reagents.

Mosquito infection rate and oocyst density were analysed at timepoints after baseline only for those individuals who were infectious at baseline. The prevalence of gametocytes and infectious individuals were compared within and between treatment groups using one-sided Fishers exact tests. Haemoglobin and methaemoglobin levels were compared using paired *t* tests (*t* score) for within-group analyses and linear regression adjusted for baseline levels of each measure for between-group analyses (*t* score, coefficient with 95% CI). Percentage change from baseline was analysed using two-way *t* tests. The proportion of gametocytes that were male was analysed for all values with total gametocyte densities of 0·2 gametocytes per μL or more.^9^ Gametocyte circulation time was calculated to determine the mean number of days that a mature gametocyte circulates in the blood before clearance, using a deterministic compartmental model that assumes a constant rate of clearance and has a random effect to account for repeated measures on individuals, as described previously;^12^ difference in circulation time between groups and between gametocyte sexes was analysed using *t* tests (*t* score), and the dose effect of tafenoquine on circulation time was determined using linear regression analysis. AUC of gametocyte density per participant over time was calculated using the linear trapezoid method,^13^ and was analysed by fitting linear regression models to the log_10_ adjusted AUC values, with adjustment for baseline gametocyte density (*t* score, coefficient with 95% CI). All other analyses of quantitative data were done using Wilcoxon sign rank tests (z-score) and Wilcoxon rank-sum tests (z-score). All comparisons were defined before study completion and analyses were not adjusted for multiple comparisons. For all analyses, the threshold for statistical significance was set at p<0·05. Statistical analysis was conducted using STATA (version 16.0) and SAS (version 9.4). The trial is registered with ClinicalTrials.gov, NCT04609098.

### Role of the funding source

The funder of the study had no role in study design, data collection, data analysis, data interpretation, or writing of the report.

## Results

**Figure 1:**
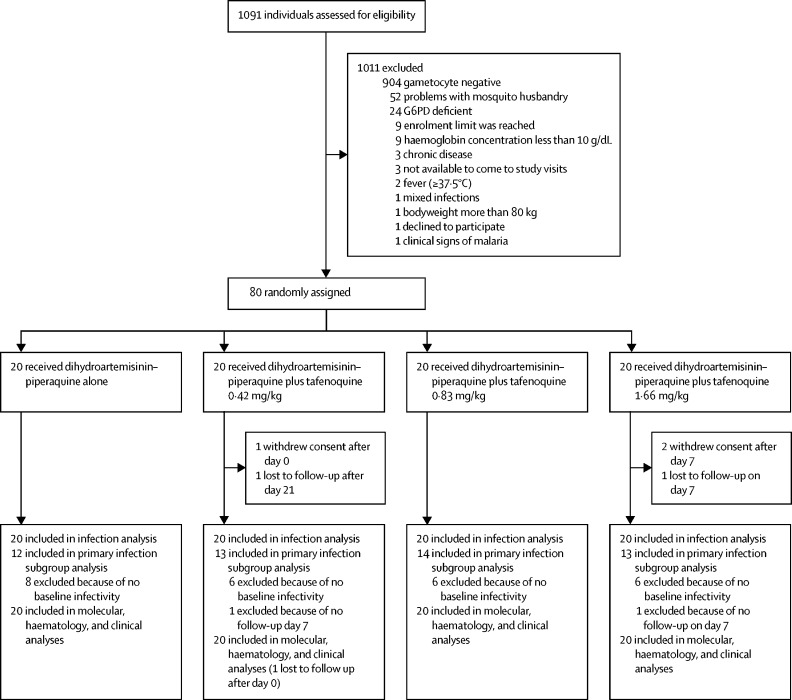
Trial profile G6PD=glucose-6-phosphate dehydrogenase.

Between Oct 29 and Nov 25, 2020, 1091 individuals were screened for eligibility, 80 of whom were enrolled and randomly assigned (20 to each treatment group; figure 1). Participant baseline characteristics were similar between the study groups, although the proportion of male participants was higher in the dihydroartemisinin–piperaquine plus tafenoquine 0·83 mg/kg and 1·66 mg/kg groups than in the dihydroartemisinin–piperaquine plus tafenoquine 0·42 mg/kg and dihydroartemisinin–piperaquine-only groups (table 1).

**Table 1:** Baseline characteristics

	**Dihydroartemisinin–piperaquine (n=20)**	**Dihydroartemisinin–piperaquine plus tafenoquine 0·42 mg/kg (n=20)**	**Dihydroartemisinin–piperaquine plus tafenoquine 0·83 mg/kg (n=20)**	**Dihydroartemisinin–piperaquine plus tafenoquine 1·66 mg/kg (n=20)**
Age, years	17 (14–19)	13 (12–17)	15 (12–32)	15 (13–21)
Female	8 (40%)	8 (40%)	6 (30%)	6 (30%)
Male	12 (60%)	12 (60%)	14 (70%)	14 (70%)
Haemoglobin, g/dL	12·6 (10·9–15·7)	12·3 (10·2–15·8)	12·4 (11·1–14·8)	12·9 (10·8–15·0)
Gametocyte prevalence	20 (100%)	20 (100%)	20 (100%)	20 (100%)
Gametocyte density, parasites per μL	41·5 (12·5–95·9)	29·5 (12·3–67·8)	43·7 (18·5–245·0)	50·1 (17·8–201·8)
Asexual parasite prevalence	12 (60%)	11 (55%)	8 (40%)	15 (75%)
Asexual parasite density, parasites per μL	120 (0–560)	60 (0–300)	0 (0–80)	500 (60–1840)

Data are median (IQR), n (%), or median (IQR). Gametocyte prevalence and density were calculated from reverse transcriptase quantitative PCR targeting CCP4/PfMGET mRNA (gametocytes). Asexual parasite prevalence and density were assessed by thick film microscopy.

**Figure 2:**
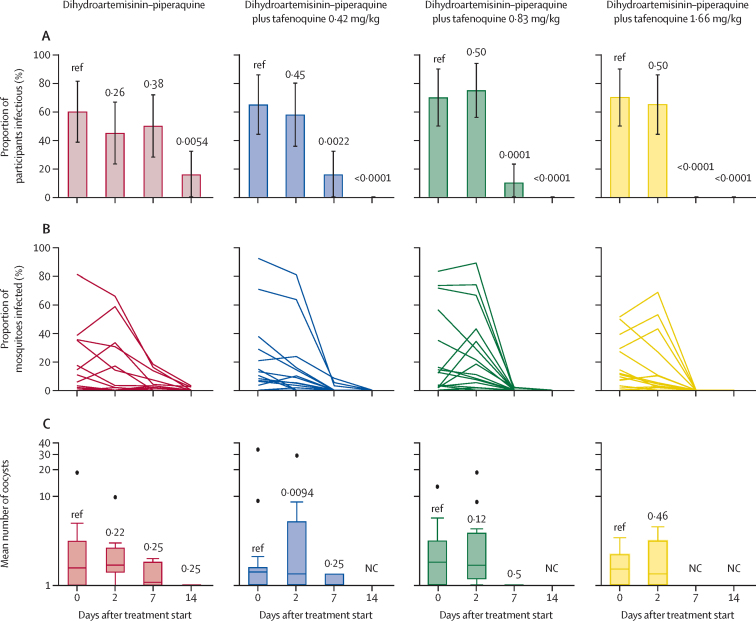
Participant infectivity, proportion of mosquitoes infected, and oocyst density in direct membrane feeding assays (A) Participant infectivity. Error bars are 95% CIs. p values from generalised linear models (family: binary) testing differences within treatment groups with baseline as reference are shown. The denominator for the proportion of infectious participants is the total number of participants still enrolled at a given timepoint, rather than the number tested at that time point for infectivity; infectivity assays were discontinued when a participant did not infect mosquitoes at two subsequent timepoints and were thereafter considered non-infectious. Full mosquito infection data including the proportion of infectious participants with denominator as total participants tested is shown in appendix 5 (p 6). (B) Mosquito infection rate. Each line represents one participant. Statistical analyses are shown in appendix 5 (p 6). (C) Oocyst density. Box plots show the median (central line), IQR (box limits), upper and lower quartiles plus 1·5 × IQR (whiskers), and outliers for mean oocyst densities in infected mosquitoes within each participant. Wilcoxon sign rank tests for differences in average oocyst density are shown. NC=not calculable.

The primary outcome measure was recorded on day 7 of follow-up, with 79 (99%) of 80 individuals completing this study visit (one in the dihydroartemisinin–piperaquine plus tafenoquine 0·42 mg/kg group did not complete the visit). 76 (95%) participants completed all visits to day 28 (two in the dihydroartemisinin–piperaquine plus tafenoquine 0·42 mg/kg group and two in the dihydroartemisinin–piperaquine plus tafenoquine 1·66 mg/kg group did not complete all visits). The median number of mosquitoes dissected in an individual mosquito feeding experiment was 55 (IQR 45–62). Before treatment, 53 (66%) individuals were infectious to mosquitoes, with a median of 12·50% (IQR 3·64–35·00)of mosquitoes becoming infected (table 2). The proportion of individuals who infected any mosquitoes at day 7 is shown in table 2 (figure 2; appendix 5 p 6). At day 7 there was a significant within-person reduction in mosquito infection rate in all four treatment groups (relative to baseline in each group), although the median reduction was greater in the three groups with tafenoquine than in the dihydroartemisinin–piperaquine-only group (table 2). At day 2, the proportion of individuals who were infectious to mosquitoes was not significantly different from baseline (figure 2; appendix 5 p 6) and the reduction in mosquito infection rate was not significant for any treatment group (table 2). The final mosquito feeds were done at day 14 after treatment, at which point three (16%) of 19 individuals in the dihydroartemisinin–piperaquine-only group were still infectious to mosquitoes, with a median mosquito infection rate of 0% (IQR 0–1·06) among individuals positive at baseline, and 2·38% (2·13–3·03) among those positive at day 14; no participants were infectious at day 14 in the tafenoquine treatment groups. Oocyst densities in infected mosquitoes were not significantly different between treatment groups at any pretreatment or post-treatment timepoint (figure 2; appendix 5 p 6). There were also no significant differences in oocyst density relative to baseline until day 7 post-treatment, at which point oocyst densities were significantly reduced in all groups (figure 2; appendix 5 p 6).

**Table 2:** Infectivity to mosquitoes before and after treatment

	Infectious individuals*	**Median mosquito infection rate**†**(IQR)**	**Median reduction in mosquito infection rate**‡**(IQR)**	**p value**§	**p value**¶
**Pretreatment**					
Dihydroartemisinin–piperaquine	12/20 (60%)	12·50% (2·44 to 35·24)	..	..	..
Dihydroartemisinin–piperaquine plus tafenoquine 0·42 mg/kg	13/20 (65%)	12·96% (6·67 to 28·79)	..	..	..
Dihydroartemisinin–piperaquine plus tafenoquine 0·83 mg/kg	14/20 (70%)	13·39% (3·03 to 56·52)	..	..	..
Dihydroartemisinin–piperaquine plus tafenoquine 1·66 mg/kg	14/20 (70%)	11·35% (6·82 to 29·17)	..	..	..
**Day 2**					
Dihydroartemisinin–piperaquine	9/20 (45%)	8·63% (0 to 31·92)	70·68% (−19·34 to 100)	0·33	Reference
Dihydroartemisinin–piperaquine plus tafenoquine 0·42 mg/kg	11/19 (58%)	9·39% (4·09 to 19·71)	36·17% (11·33 to 54·25)	0·063	0·50
Dihydroartemisinin–piperaquine plus tafenoquine 0·83 mg/kg	15/20 (75%)	19·77% (7·35 to 43·08)	15·35% (−100 to 48·96)	1·00	0·094
Dihydroartemisinin–piperaquine plus tafenoquine 1·66 mg/kg	13/20 (65%)	7·59% (2·38 to 26·79)	51·23% (−36·67 to 68·63)	0·12	0·58
**Day 7**					
Dihydroartemisinin–piperaquine	10/20 (50%)	2·36% (0 to 10·39)	79·95% (57·15 to 100)	0·0005	Reference
Dihydroartemisinin–piperaquine plus tafenoquine 0·42 mg/kg	3/19 (16%)	0% (0 to 1·52)	100% (98·36 to 100)	0·0005	0·020
Dihydroartemisinin–piperaquine plus tafenoquine 0·83 mg/kg	2/20 (10%)	0% (0 to 0)	100% (100 to 100)	0·0001	0·011
Dihydroartemisinin–piperaquine plus tafenoquine 1·66 mg/kg	0/19	0% (0 to 0)	100% (100 to 100)	0·0001	0·0006
**Day 14**					
Dihydroartemisinin–piperaquine	3/19 (16%)	0% (0 to 1·06)	0% (0 to 1·06)	0·0005	Reference
Dihydroartemisinin–piperaquine plus tafenoquine 0·42 mg/kg	0/19	0% (0 to 0)	0% (0 to 0)	0·0005	0·22
Dihydroartemisinin–piperaquine plus tafenoquine 0·83 mg/kg	0/19	0% (0 to 0)	0% (0 to 0)	0·0001	0·17
Dihydroartemisinin–piperaquine plus tafenoquine 1·66 mg/kg	0/17	0% (0 to 0)	0% (0 to 0)	0·0005	0·22

* Individuals were classed as infectious if direct membrane feeding assays resulted in at least one mosquito with any number of oocysts. Mosquito infection measures (percentage infection and oocyst density) are shown for all participants who were infectious at baseline, and oocyst densities are from all infected mosquitoes.

† Median proportion of mosquitoes infected by each participant, where for each participant the mosquito infection rate was the number of mosquitoes infected as a proportion of all mosquitoes surviving to dissection.

‡ Median reduction (relative to baseline) in mosquito infection rate at the given timepoints. All values are for individuals who were infectious to mosquitoes before treatment (ie, infected any number of mosquitoes).

§ Within-group comparison in median reduction in mosquito infection rate (primary outcome).

¶ Between-group comparison in median reduction in mosquito infection rate. Full details of mosquito feeding assay outcomes are in appendix 5 (pp 6–7).

**Figure 3:**
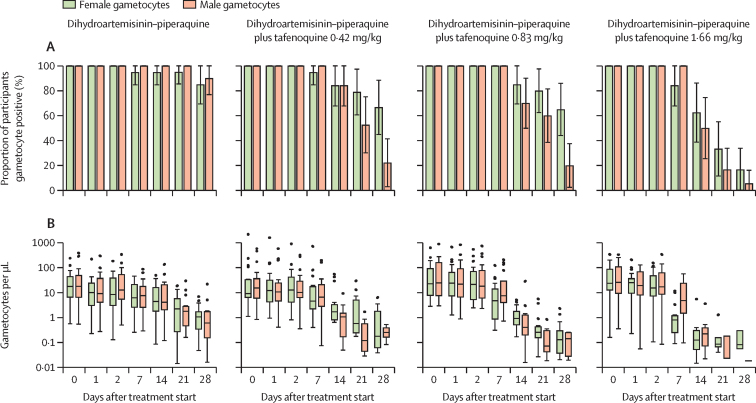
Male and female gametocyte density and prevalence (A) Gametocyte prevalence. Error bars are 95% CIs. (B) Gametocyte density, shown for gametocyte positive individuals only (ie, male or female density >0·01 per μL). Box plots show the median (central line), IQR (box limits), upper and lower quartiles plus 1·5 × IQR (whiskers), and outliers. Within and between group statistical analyses of gametocyte density and prevalence are shown in appendix 5 (p 8). Gametocyte circulation time, area under the curve, and other outcomes are shown in appendix 5 (pp 7–11).

Gametocyte densities decreased after initiation of treatment in all study groups, although the decrease was more rapid in the tafenoquine groups than in the dihydroartemisinin–piperaquine-only group (figure 3; appendix 5 pp 8–9). Total gametocyte prevalence by the final day of follow-up (day 28) was 18 (90%) of 20 participants in the dihydroartemisinin–piperaquine-only group, 12 (67%) of 18 participants in the dihydroartemisinin–piperaquine plus tafenoquine 0·42 mg/kg group, 13 (65%) of 20 participants in the dihydroartemisinin–piperaquine plus tafenoquine 0·83 mg/kg group, and three (17%) of 18 in the dihydroartemisinin–piperaquine plus tafenoquine 1·66 mg/kg group. Total gametocyte circulation time was estimated at 8·3 days (95% CI 7·0–9·6) in the dihydroartemisinin–piperaquine-only group, and decreased to 2·7 days (2·5–2·9) in the dihydroartemisinin–piperaquine plus tafenoquine 1·66 mg/kg group. We observed evidence for a dose–response effect of tafenoquine on gametocyte clearance; doubling the tafenoquine dose was associated with a decrease in circulation time of 1·2 days (0·9–1·5) for female gametocytes and 0·5 days (0·3–0·7) for male gametocytes. Gametocyte AUC was similarly lower in the tafenoquine treatment groups than in the dihydroartemisinin–piperaquine-only group, after adjustment for baseline gametocyte density (appendix 5 p 7). Gametocyte sex ratios were initially similar in all treatment groups (median proportion male 0·51 [IQR 0·42–0·62]) but female gametocyte density decreased substantially in the dihydroartemisinin–piperaquine plus tafenoquine 1·66 mg/kg group by day 7 after treatment, resulting in significantly male-biased sex ratios (appendix 5 pp 8, 10). From day 14, total gametocyte densities became too low to reliably determine sex ratio in the majority of samples in the dihydroartemisinin–piperaquine plus tafenoquine 1·66 mg/kg group (eight samples remained with densities >0·2 per μL). At day 7, the infectivity of persisting gametocytes was significantly lower in the tafenoquine treatment groups compared with the dihydroartemisinin treatment group (appendix 5 p 8).

There was a small but statistically significant within-group reduction in haemoglobin density in the dihydroartemisinin–piperaquine-only and dihydroartemisinin–piperaquine plus tafenoquine 0·83 mg/kg groups at days 2 and 7 compared with baseline, with no significant reductions in any other treatment group or at any other timepoint (appendix 5 pp 11, 13). The greatest mean reduction in haemoglobin density in any treatment group or timepoint was −3·3% (95% CI −4·7 to −1·8) in the dihydroartemisinin–piperaquine-only group at day 7 (appendix 5 p 11). The greatest reduction in haemoglobin density in any individual was 25·6% (from 13·3 g/dL at baseline to 9·9 g/dL at day 21 in an individual in the dihydroartemisinin–piperaquine plus tafenoquine 1·66 mg/kg group). This was the lowest observed haemoglobin density in any individual and timepoint and increased to 10·3 g/dL at the next visit. Methaemoglobin concentration was significantly increased from baseline between days 7 and 21 in the dihydroartemisinin–piperaquine plus tafenoquine 0·83 mg/kg and 1·66 mg/kg groups, but was only significantly greater than the dihydroartemisinin–piperaquine-only group in the dihydroartemisinin–piperaquine plus tafenoquine 1·66 mg/kg group (at all timepoints other than baseline and day 21; appendix 5 pp 12–13).

Overall, 55 (69%) of 80 participants had a total of 94 adverse events during follow-up, of which 86 (92%) were categorised as mild, seven (7%) as moderate, and one (1%) as severe (appendix 5 pp 14–15). The single severe adverse event was a urinary tract infection not related to the study in a 25-year-old woman in the dihydroartemisinin–piperaquine plus tafenoquine 1·66 mg/kg group at day 7. This infection was resolved without sequela with appropriate treatment. There was no difference between treatment groups in the proportion of participants who had any adverse event (p=0·73), or adverse events of mild (p=0·95) or moderate (p=0·75) severity. The most common treatment-related adverse event was mild or moderate headache, which occurred in 15 (19%) participants (dihydroartemisinin–piperaquine n=2; dihydroartemisinin–piperaquine plus tafenoquine 0·42 mg/kg n=6; dihydroartemisinin–piperaquine plus tafenoquine 0·83 mg/kg n=3; and dihydroartemisinin–piperaquine plus tafenoquine 1·66 mg/kg n=4). No serious adverse events occurred. There was no difference between treatment groups in the proportion of participants who had any adverse event that was possibly, probably, or definitely related to treatment (p=0·62; appendix 5 p 14). No individuals had clinically significant creatinine, aspartate aminotransferase, or alanine aminotransferase concentrations outside the normal range after treatment. In the dihydroartemisinin–piperaquine plus tafenoquine 0·42 mg/kg group, two individuals had transient increases in alanine aminotransferase outside the normal range, categorised as mild adverse events, on day 2 and 7, and one individual had a transient increase in creatine at day 7, categorised as a moderate adverse event. All were classified as possibly drug related, and all normalised on the subsequent visit (appendix 5 p 16).

## Discussion

To our knowledge, this was the first clinical trial specifically designed to determine the *P falciparum* gametocytocidal and transmission-blocking properties of single low doses of tafenoquine. By day 7, post-treatment transmission potential was greatly reduced in the 0·42 and 0·83 mg/kg tafenoquine dose groups, and completely annulled in individuals given the highest (1·66 mg/kg) tafenoquine dose.

ACTs differ in their gametocytocidal properties.^14^ Gametocyte clearance by antimalarial drugs might be preceded by distortions in gametocyte sex ratio or gametocyte fitness that prevent onward transmission to mosquitoes,^15^, ^16^ highlighting the value of true assessment of infectivity through mosquito feeding assays when examining a drug's transmission-reducing properties. The findings from our study support earlier reports of persisting gametocyte carriage and transmission in the weeks following treatment with dihydroartemisinin–piperaquine.^9^, ^11^ In the current study, gametocyte carriage persisted in all individuals treated with dihydroartemisinin–piperaquine-only until the end of follow-up (day 28) and three (16%) of 19 individuals were still infectious to mosquitoes 14 days after initiation of treatment. The addition of tafenoquine accelerated clearance of both male and female gametocytes in a dose-dependent manner; this is in line with reported in-vitro *P falciparum* gametocytocidal activity of tafenoquine.^5^ Comparing the current findings with previous trials on the transmission-blocking effects of primaquine, wherein primaquine blocked transmission by day 2 post-treatment,^9^, ^11^ gametocyte clearance appears to be slower after tafenoquine, corroborating findings from a clinical trial in patients with *P vivax* in which tafenoquine had a longer time to gametocyte and parasite clearance compared with chloroquine plus primaquine.^17^ Contrary to observations with primaquine,^9^, ^18^ we also observed no evidence for an early sterilising effect of tafenoquine. Only by day 7 did we observe lower per-gametocyte infectivity in the tafenoquine treatment groups that we interpret as an indication that some gametocytes lose viability before their clearance; studies with more observations before day 7 are required to examine this further.^15^ Our observation that the transmission-blocking activity of tafenoquine occurs later than day 2 is in line with findings from an avian *Plasmodium gallinaceum* model in which transmission to mosquitoes persisted for 4 days after initiation of tafenoquine monotherapy treatment and declined thereafter.^19^ The reasons for this slow effect are unclear and could be related to both tafenoquine metabolism and activity. There is no consensus on the importance of CYP2D6-dependent tafenoquine activation: although rodent models indicate a relevant role for CYP2D6,^20^, ^21^ CYP2D6 intermediate metabolisers did not show lower tafenoquine activity against *P vivax* relapses^22^ and CYP2D6-mediated activation thus appears less important for tafenoquine than for primaquine. Tafenoquine's activity against schizonts appears to be CYP2D6 independent,^23^ but this does not rule out the possibility of CYP2D6 dependence for the drug's gametocytocidal activity. A generally lower activity of tafenoquine that only takes effect after several days of drug exposure might explain the late transmission-blocking properties of tafenoquine. More frequent assessments of infectivity in the first week after treatment would allow refinement of time of action.

Despite not having a direct comparison to dihydroartemisinin–piperaquine plus single low-dose primaquine, the current study suggests inferior transmission-blocking properties of the tested doses of tafenoquine compared with single low-dose primaquine tested in similar populations in multiple recent studies. Nonetheless, tafenoquine's long half-life could be a major advantage. Depending on the duration of its gametocytocidal activity, tafenoquine might play a role in preventing transmission of infections that are acquired after initiation of treatment. A long-acting gametocytocide could also be of relevance to prevent the transmission of drug-resistant parasites. Treatment failure has been associated with increased appearance of gametocytes after initiation of treatment,^24^, ^25^ and artemisinin-resistant gametocytes might have enhanced transmission potential under drug pressure.^26^ A short-acting gametocytocidal drug such as primaquine delivered as a single dose is unlikely to affect gametocytes that appear in circulation after initiation of treatment, whereas tafenoquine might plausibly clear these gametocytes and thereby prevent transmission of gametocytes related to delayed parasite clearance or recrudescence.

Whether low-dose gametocytocides are included in standard treatment or mass administrations, the choice of partner schizonticide requires careful consideration. The current study shows that dihydroartemisinin–piperaquine plus single low-dose tafenoquine has substantial gametocytocidal activity at doses of 1·66 mg/kg, but it is noteworthy that preliminary observations from an unpublished *P vivax* treatment trial indicate that that dihydroartemisinin–piperaquine might inhibit tafenoquine activity against *P vivax* hypnozoites.^27^ Tafenoquine doses for *P vivax* are only recommended for use with chloroquine.^28^ Given that chloroquine resistance is widespread for *P falciparum*, it seems logical that single low-dose tafenoquine will need to be combined with ACT. If the safety of these tafenoquine doses in G6PD mixed populations can be demonstrated, our data indicate this combination will be effective at clearing asexual parasites and transmission blockade.

To be used widely, tafenoquine's safety is a key consideration. We observed no increased number of adverse events in tafenoquine treatment groups compared with dihydroartemisinin–piperaquine-only. Non-symptomatic transient increases in alanine aminotransferase were observed in two individuals who received the lowest tafenoquine dose; this might be associated with general infection-related liver-injury that is treatment independent.^29^ Due to the potentially greater risk of haemolysis in G6PD-deficient individuals,^30^ our efficacy study population consisted only of adults with normal G6PD production. The distribution of G6PD deficiency worldwide is similar to that of malaria.^31^ It is therefore essential that the minimally efficacious dose and safety thereof in individuals with normal G6PD production is established. No significant decreases in haemoglobin or increase in adverse events were observed in the tafenoquine groups compared with the dihydroartemisinin–piperaquine-only group. Moreover, the tafenoquine doses tested in our study population appear no less safe than single low-dose primaquine tested at the same location and with a similar population (ie, identical inclusion and exclusion criteria).^9^ Although testing for G6PD deficiency is presently required before administration of tafenoquine for its current indications, future studies should explore the safety profile of single low doses of tafenoquine in larger populations that include participants who are G6PD deficient to inform the highest tolerable dose without previous G6PD testing. These studies should include a single low-dose comparator group. Future studies should also be conducted in younger populations. Two previous studies with tafenoquine have been done in children. A study in Gabon with various tafenoquine doses for prophylaxis in semi-immune children and young adults between 12 and 20 years of age reported similar adverse events between treatment groups and compared with the placebo group but an average reduction in haemoglobin concentrations compared with baseline of 0·4 mg/dL with the highest tafenoquine dose of 200 mg.^32^ Another phase 2 study assessed the safety and efficacy of chloroquine plus single weight-dependent doses of tafenoquine (100 mg, 150 mg, 200 mg, or 300 mg) in children aged between 2 and 15 years with *P vivax* malaria and G6PD enzyme activity of at least 70% in Columbia and Vietnam.^33^ The most common adverse events attributed to drug treatment were vomiting and asymptomatic methaemoglobin increases. Haematological safety of tafenoquine in this study was similar to studies with (young) adults.

In the current study, we established the short-term effect of tafenoquine on transmission from highly infectious individuals. This population allows a detailed assessment of tafenoquine's transmission-blocking properties with high discriminative power. Our study shows that a dose of 1·66 mg/kg tafenoquine blocks transmission by day 7 but this effect might have been apparent at earlier moments when we did not do feeding experiments (days 3–7). In addition, our study provides no evidence for the duration of tafenoquine's transmission blocking activity. Future trials are required to examine the timing of tafenoquine activity with respect to both the initiation and duration of its *P falciparum* gametocytocidal activity. A design with a follow-up over multiple malaria episodes or in which tafenoquine is administered without an efficacious schizonticidal drug would allow assessment of the duration of tafenoquine's activity. With increasing evidence for the transmission-blocking effects of primaquine and tafenoquine in individual gametocyte carriers, there is an urgent need for community trials to establish the added value of these gametocytocides in first-line antimalarial treatment and in mass treatment campaigns to reduce the transmission of (drug-resistant) malaria.

## Data sharing

Anonymised data reported in the manuscript will be made available to investigators who provide a methodologically sound proposal to the corresponding author. The protocol is available upon request.

## Declaration of interests

We declare no competing interests.

## Supplementary Materials

French translation of the abstract

Portuguese translation of the abstract

Spanish translation of the abstract

Swahili translation of the abstract
