# Correction to *Lancet Microbe* 2022; 3: e336–47

**DOI:** 10.1016/S2666-5247(22)00228-2

**Published:** 2022-10

*Stone W, Mahamar A, Smit MJ, et al. Single low-dose tafenoquine combined with dihydroartemisinin–piperaquine to reduce* Plasmodium falciparum *transmission in Ouelessebougou, Mali: a phase 2, single-blind, randomised clinical trial.* Lancet Microbe *2022; **3:** e336–47*—In this Article, the date of the final follow-up visit in the final sentence of the Methods section in the Summary should have read “Dec 23, 2020”. This correction has been made as of Aug 11, 2022.

