# Supplementary material for: Single low-dose tafenoquine combined with dihydroartemisinin–piperaquine to reduce Plasmodium falciparum transmission in Ouelessebougou, Mali: a phase 2, single-blind, randomised clinical trial
Source: Lancet Microbe. 2022 May;3(5):e336–47. doi: 10.1016/S2666-5247(21)00356-6 (PMC9042793; doi:10.1016/S2666-5247(21)00356-6)
Supplement: French translation of the abstract [file mmc1.pdf]

THE LANCET
Microbe

Supplementary appendix 1

This translation in French was submitted by the authors and we reproduce it as supplied. It has not been peer reviewed. *The Lancet*'s editorial processes have only been applied to the original in English, which should serve as reference for this manuscript.

Supplement to: Stone W, Mahamar A, Smit MJ, et al. Single low-dose tafenoquine combined with dihydroartemisinin–piperaquine to reduce *Plasmodium falciparum* transmission in Ouelessebougou, Mali: a phase 2, single-blind, randomised clinical trial. *Lancet Microbe* 2022; published online March 23. https://doi.org/10.1016/S2666-5247(21)00356-6.

Cette traduction en français a été proposée par les auteurs et nous l'avons reproduite telle quelle. Elle n'a pas été examinée par des pairs. Les processus éditoriaux du *Lancet* n'ont été appliqués qu'à l'original en anglais et c'est cette version qui doit servir de référence pour ce manuscrit.

Résumé

Contexte

La tafenoquine a récemment été approuvée comme traitement prophylactique et curatif radical de l'infection par Plasmodium vivax, mais son efficacité pour bloquer la transmission de Plasmodium falciparum n'est pas claire. Nous avons cherché à établir l'efficacité et la sécurité de trois faibles doses uniques de tafenoquine en association avec la dihydroartémisinine-pipéraquine pour réduire la densité des gamétocytes et la transmission aux moustiques.

Méthodes

Dans cet essai contrôlé randomisé de phase 2, en simple aveugle et à quatre bras, les participants ont été recrutés à l'Unité de recherche clinique du Centre de recherche et de formation sur le paludisme de l'Université de Bamako au Mali. Les participants éligibles étaient âgés de 12 à 50 ans, présentaient un portage gamétocytaire asymptomatique détecté par microscopie à P falciparum, avaient un poids corporel de 80 kg ou moins et ne présentaient aucun signe clinique de paludisme défini par la fièvre. Les participants ont été assignés au hasard (1:1:1:1) à un traitement standard par dihydroartémisinine-pipéraquine, ou par dihydroartémisinine-pipéraquine plus une dose unique de tafenoquine (en solution) à une dose finale de 0-42 mg/kg, 0-83 mg/kg, ou 1-66 mg/kg. La randomisation a été effectuée à l'aide d'une liste de randomisation générée par ordinateur et dissimulée dans des enveloppes opaques et scellées. La dihydroartémisinine-pipéraquine a été administrée sous forme de comprimés oraux pendant 3 jours (jours 0, 1 et 2), conformément aux instructions du fabricant. Une dose unique de tafenoquine a été administrée sous forme de solution orale le jour 0, parallèlement à la première dose de dihydroartémisinine-pipéraquine. La dose de tafenoquine était basée sur le poids corporel afin de standardiser l'efficacité et la variance du risque. Le critère d'évaluation principal, évalué dans la population per protocole, était la variation médiane en pourcentage du taux d'infection par les moustiques 7 jours après le traitement par rapport à la ligne de base. Les critères d'évaluation de la sécurité comprenaient la fréquence et l'incidence des événements indésirables. La dernière visite de suivi a eu lieu le 23 décembre 2021 ; l'essai est enregistré auprès de ClinicalTrials.gov, NCT04609098.

Constatations

Du 29 octobre au 25 novembre 2020, 1091 individus ont été examinés pour leur éligibilité, 80 d'entre eux ont été inscrits et assignés au hasard (20 par groupe de traitement). Avant le traitement, 53 (66%) individus étaient infectieux pour les moustiques, infectant en médiane 12-50% des moustiques (IQR 3-64-35-00). La réduction intra-groupe du taux d'infection des moustiques au jour 7 était de 79-95% (IQR 57-15-100 ; p=0-0005 pour la différence avec la ligne de base) après le traitement par dihydroartémisinine-pipéraquine uniquement, 100% (98-36-100 ; p=0-0005) après l'administration de dihydroartémisinine-pipéraquine et de tafénoquine à raison de 0-42 mg/kg, 100 % (100-100 ; p=0-0001) après l'administration de dihydroartémisinine-pipéraquine et de tafénoquine à raison de 0-83 mg/kg, et 100 % (100-100 ; p=0-0001) après l'administration de dihydroartémisinine-pipéraquine et de tafénoquine à raison de 1-66 mg/kg. 55 (69 %) des 80 participants ont présenté un total de 94 événements indésirables au cours de l'essai ; 86 (92 %) événements indésirables ont été classés comme légers, sept (7 %) comme modérés et un (1 %) comme grave. L'événement indésirable lié au traitement le plus fréquent était la céphalée légère ou modérée, qui est survenue chez 15 (19 %) participants (dihydroartémisinine-pipéraquine n=2 ; dihydroartémisinine-pipéraquine plus tafenoquine 0-42 mg/kg n=6 ; dihydroartémisinine-pipéraquine plus tafenoquine 0-83 mg/kg n=3 ; et dihydroartémisinine-pipéraquine plus tafenoquine

1-66 mg/kg n=4). Aucun événement indésirable grave n'est survenu. Aucune différence significative dans l'incidence de tous les événements indésirables (p=0-73) ou des événements indésirables liés au traitement (p=0-62) n'a été observée entre les groupes de traitement.

Interprétation

La tafenoquine a été bien tolérée à toutes les doses et a accéléré la clairance des gamétocytes de P falciparum. Toutes les doses de tafenoquine ont montré une meilleure réduction de la transmission au jour 7 par rapport à la dihydroartémisinine-pipéraquine seule. Ces données plaident en faveur de la poursuite des recherches sur la tafenoquine en tant que complément aux antipaludéens standard pour bloquer la transmission.
