# Supplementary material for: Single low-dose tafenoquine combined with dihydroartemisinin–piperaquine to reduce Plasmodium falciparum transmission in Ouelessebougou, Mali: a phase 2, single-blind, randomised clinical trial
Source: Lancet Microbe. 2022 May;3(5):e336–47. doi: 10.1016/S2666-5247(21)00356-6 (PMC9042793; doi:10.1016/S2666-5247(21)00356-6)
Supplement: Portuguese translation of the abstract [file mmc2.pdf]

THE LANCET
Microbe

Supplementary appendix 2

This translation in Portuguese was submitted by the authors and we reproduce it as supplied. It has not been peer reviewed. *The Lancet*'s editorial processes have only been applied to the original in English, which should serve as reference for this manuscript.

Supplement to: Stone W, Mahamar A, Smit MJ, et al. Single low-dose tafenoquine combined with dihydroartemisinin–piperaquine to reduce *Plasmodium falciparum* transmission in Ouelessebougou, Mali: a phase 2, single-blind, randomised clinical trial. *Lancet Microbe* 2022; published online March 23. https://doi.org/10.1016/S2666-5247(21)00356-6.

Esta tradução em português foi submetida pelos autores e nós não fizemos quaisquer alterações. Esta versão não foi revista por pares. O processo editorial do The Lancet só foi aplicado à versão original em inglês, que deve servir como referência para este artigo.

Resumo

Antecedentes

A tafenoquina foi recentemente aprovada como profilaxia e cura radical para a infecção por *Plasmodium vivax*, mas a sua eficácia de bloqueio da transmissão por *Plasmodium falciparum* não é clara. O nosso objectivo foi estabelecer a eficácia e segurança de três doses únicas e baixas de tafenoquina em combinação com diidroartemisina-piperaquina, para reduzir a densidade de gametócitos e a transmissão aos mosquitos.

Métodos

Neste ensaio controlado, ”single-blind”, fase 2, aleatorio, os participantes foram recrutados na Unidade de Investigação Clínica do Centro de Investigação e Formação em Malária da Universidade de Bamako, no Mali. Os participantes elegíveis tinham entre 12 e 50 anos de idade, eram asintomaticos e apresentavam gametocitos de *P. falciparum* por deteccao ao microscopio, tinham um peso corporal de 80 kg ou menos, e não apresentavam sinais clínicos de malária definidos por febre. Os participantes foram escolhidos aleatoriamente (1:1:1:1) para o tratamento padrão com diidroartemisina-piperaquina, ou o tratamento com diidroartemisina-piperaquina mais uma dose única de tafenoquina (em solução) numa dose final de 0.42 mg/kg, 0.83 mg/kg, ou 1.66 mg/kg. A aleatorização foi feita com base numa lista de aleatorização gerada por computador e ocultada com envelopes selados e opacos. Diidroartemisina-piperaquina foi administrada por via oral com comprimidos durante 3 dias (dia 0, 1, e 2), de acordo com as instruções do fabricante. Uma única dose de tafenoquina foi administrada como solução oral no dia 0, em paralelo com a primeira dose de diidroartemisina-piperaquina. A dose de tafenoquina foi baseada no peso corporal para normalizar a eficácia e a variação do risco. O principal criterio de avaliacao avaliado na população foi a variação percentual mediana da taxa de infecção por mosquito 7 dias após o tratamento, em comparação com a linha de base. Os parâmetros de segurança incluíram a frequência e incidência de eventos adversos. A visita final de seguimento foi em 23 de Dezembro de 2021; o ensaio está registado no ClinicalTrials.gov, NCT04609098.

Descobertas

De 29 de Outubro a 25 de Novembro de 2020, 1091 indivíduos foram seleccionados para elegibilidade, 80 dos quais foram inscritos e distribuídos aleatoriamente (20 por grupo de tratamento). Antes do tratamento, 53 (66%) indivíduos mostraram-se infecciosos aos mosquitos, infectando uma mediana de 12-50% dos mosquitos (IQR 3-64-35-00). A redução da taxa de infecção por mosquitos dentro do grupo no dia 7 foi de 79-95% (IQR 57-15-100; p=0-0005 para a diferença em relação à linha de base) apenas após a diidroartemisina-piperaquina, 100% (98-36-100; p=0-0005) a seguir à di-hidroartemisina-piperaquina mais tafenoquina 0.42 mg/kg, 100% (100-100; p=0-0001) a seguir à di-hidroartemisina-piperaquina mais tafenoquina 0.83 mg/kg, e 100% (100-100; p=0-0001) a seguir à di-hidroartemisina-piperaquina mais tafenoquina 1.66 mg/kg. 55 (69%) dos 80 participantes tiveram um total de 94 acontecimentos adversos durante o ensaio; 86 (92%) acontecimentos adversos foram classificados como leves, sete (7%) como moderados, e um (1%) como severos. O evento adverso mais comum relacionado com o tratamento foi a dor de cabeça leve ou moderada, que ocorreu em 15 (19%) dos participantes (diidroartemisina-piperaquina n=2; diidroartemisina- piperaquina mais tafenoquina 0.42 mg/kg n=6; diidroartemisina-piperaquina mais tafenoquina 0.83 mg/kg n=3; e diidroartemisina-piperaquina mais tafenoquina 1.66 mg/kg n=4). Não ocorreram acontecimentos adversos graves. Não foram observadas diferenças significativas na

incidência de todos os acontecimentos adversos (p=0-73) ou acontecimentos adversos relacionados com o tratamento (p=0-62) entre os grupos de tratamento.

Interpretação

A tafenoquina foi bem tolerada em todas as doses e acelerou a eliminação de gametócitos de *P. falciparum*. Todas as doses de tafenoquina mostraram uma melhor redução da transmissão no 7º dia em comparação apenas com a diidroartemisina-piperaquina. Estes dados apoiam a necessidade de mais investigação sobre a tafenoquina como suplemento de bloqueio de transmissão dos anti-maláricos padrão.
