# Supplementary material for: Single low-dose tafenoquine combined with dihydroartemisinin–piperaquine to reduce Plasmodium falciparum transmission in Ouelessebougou, Mali: a phase 2, single-blind, randomised clinical trial
Source: Lancet Microbe. 2022 May;3(5):e336–47. doi: 10.1016/S2666-5247(21)00356-6 (PMC9042793; doi:10.1016/S2666-5247(21)00356-6)
Supplement: Spanish translation of the abstract [file mmc3.pdf]

THE LANCET
Microbe

Supplementary appendix 3

This translation in Spanish was submitted by the authors and we reproduce it as supplied. It has not been peer reviewed. *The Lancet*'s editorial processes have only been applied to the original in English, which should serve as reference for this manuscript.

Supplement to: Stone W, Mahamar A, Smit MJ, et al. Single low-dose tafenoquine combined with dihydroartemisinin–piperaquine to reduce *Plasmodium falciparum* transmission in Ouelessebougou, Mali: a phase 2, single-blind, randomised clinical trial. *Lancet Microbe* 2022; published online March 23. https://doi.org/10.1016/S2666-5247(21)00356-6.

Los autores nos proporcionaron esta traducción al español y la reproducimos tal como nos fue entregada. No la hemos revisado. Los procesos editoriales de *The Lancet* se han aplicado únicamente al original en inglés, que debe servir de referencia para este manuscrito.

Resumen

Antecedentes

La tafenoquina fue aprobada recientemente como profilaxis y cura radical de la infección por Plasmodium vivax, pero su eficacia para bloquear la transmisión de Plasmodium falciparum no está clara. Nuestro objetivo es establecer la eficacia y seguridad de tres dosis bajas únicas de tafenoquina en combinación con dihidroartemisinina-piperaquina para reducir la densidad de gametocitos y la transmisión a los mosquitos.

Métodos

En este ensayo controlado aleatorizado simple ciego de fase 2, y con cuatro brazos, los participantes fueron reclutados en la Unidad de Investigación Clínica del Centro de Investigación y Formación sobre el Paludismo de la Universidad de Bamako (Mali). Los participantes elegibles tenían entre 12 y 50 años, eran portadores asintomáticos de gametocitos de P falciparum detectados por microscopía, tenían un peso corporal de 80 kg o menos y no presentaban signos clínicos de malaria definidos por fiebre. Los participantes fueron asignados aleatoriamente (1:1:1:1) a un tratamiento estándar con dihidroartemisina-piperaquina, o dihidroartemisina-piperaquina más una dosis única de tafenoquina (en solución) a una dosis final de 0-42 mg/kg, 0-83 mg/kg, o 1-66 mg/kg. La aleatorización se realizó con una lista de aleatorización generada por ordenador y se enmascaró con sobres opacos sellados. La dihidroartemisina-piperaquina se administró en forma de comprimidos orales durante 3 días (día 0, 1 y 2), según las instrucciones del fabricante. Se administró una dosis única de tafenoquina en forma de solución oral el día 0 en paralelo a la primera dosis de dihidroartemisina-piperaquina. La dosificación de la tafenoquina se basó en el peso corporal para estandarizar la eficacia y la variación del riesgo. El criterio de evaluación primario, evaluado en la población por protocolo, fue el cambio porcentual de la mediana en la tasa de infección por mosquitos 7 días después del tratamiento en comparación con el inicio. Los criterios de evaluación de la seguridad incluyeron la frecuencia y la incidencia de acontecimientos adversos. La última visita de seguimiento fue el 23 de diciembre de 2021; el ensayo está registrado en ClinicalTrials.gov, NCT04609098.

Hallazgos

Entre el 29 de octubre y el 25 de noviembre de 2020, se examinaron 1091 individuos para determinar su elegibilidad, 80 de los cuales fueron inscritos y asignados al azar (20 por grupo de tratamiento). Antes del tratamiento, 53 (66%) individuos fueron infecciosos para los mosquitos, infectando una mediana de 12-50% de los mosquitos (IQR 3-64-35-00). La reducción dentro de cada grupo de la tasa de infección por mosquitos en el día 7 fue del 79-95% (IQR 57-15-100; p=0-0005 para la diferencia con respecto a la línea de base) después de la dihidroartemisinina-piperaquina solamente, el 100% (98-36-100; p=0-0005) tras dihidroartemisina-piperaquina más tafenoquina 0-42 mg/kg, 100% (100-100; p=0-0001) tras dihidroartemisina-piperaquina más tafenoquina 0-83 mg/kg, y 100% (100-100; p=0-0001) tras dihidroartemisina-piperaquina más tafenoquina 1-66 mg/kg. 55 (69%) de los 80 participantes tuvieron un total de 94 acontecimientos adversos en el transcurso del ensayo; 86 (92%) acontecimientos adversos se clasificaron como leves, siete (7%) como moderados y uno (1%) como grave. El acontecimiento adverso más frecuente relacionado con el tratamiento fue el dolor de cabeza leve o moderado, que se produjo en 15 (19%) participantes (dihidroartemisina-piperaquina n=2; dihidroartemisina-piperaquina más tafenoquina 0-42 mg/kg n=6; dihidroartemisina-piperaquina más tafenoquina 0-83 mg/kg n=3; y dihidroartemisina-piperaquina más tafenoquina 1-66 mg/kg n=4). No se produjeron acontecimientos adversos graves. No se observaron diferencias significativas en la incidencia de todos los acontecimientos adversos (p=0-73)

o de los acontecimientos adversos relacionados con el tratamiento (p=0-62) entre los grupos de tratamiento.

Interpretación

La tafenoquina fue bien tolerada en todas las dosis y aceleró la eliminación de gametocitos de P falciparum. Todas las dosis de tafenoquina mostraron una mejor reducción de la transmisión en el día 7 en comparación con la dihidroartemisinina-piperaquina sola. Estos datos respaldan la necesidad de seguir investigando la tafenoquina como complemento de los antimaláricos estándar para bloquear la transmisión.
