# Supplementary material for: Single low-dose tafenoquine combined with dihydroartemisinin–piperaquine to reduce Plasmodium falciparum transmission in Ouelessebougou, Mali: a phase 2, single-blind, randomised clinical trial
Source: Lancet Microbe. 2022 May;3(5):e336–47. doi: 10.1016/S2666-5247(21)00356-6 (PMC9042793; doi:10.1016/S2666-5247(21)00356-6)
Supplement: Swahili translation of the abstract [file mmc4.pdf]

THE LANCET
Microbe

Supplementary appendix 4

This translation in Swahili was submitted by the authors and we reproduce it as supplied. It has not been peer reviewed. *The Lancet*'s editorial processes have only been applied to the original in English, which should serve as reference for this manuscript.

Supplement to: Stone W, Mahamar A, Smit MJ, et al. Single low-dose tafenoquine combined with dihydroartemisinin–piperaquine to reduce *Plasmodium falciparum* transmission in Ouelessebougou, Mali: a phase 2, single-blind, randomised clinical trial. *Lancet Microbe* 2022; published online March 23. https://doi.org/10.1016/S2666-5247(21)00356-6.

Tafsiri hii katika Swahili iliwasilishwa na waandishi na tunatengeneza tena kama hutolewa. Haijapitiwa. Mchakato wa hariri wa Lancet Global Health umetumika tu kwa asili kwa Kiingereza, ambayo inapaswa kutumika kama kumbukumbu kwa muswada hii.

Muhtasari

Utangulizi

Tefenoquine imeidhinishwa hivi karibuni kutumika kama dawa ya kinga na tiba ya awali ya uambukizo wa Plasmodium vivax, lakini uwezo wake wa kuzuia kusambaa kwa Plasmodium falciparum haijabainishwa. Tulidhamiria kuanza kuangalia uwezo na usalama wa dozi ndogo tatu za mara moja za tafenoquine ikichanganywa na dihydroartemisinin–piperaquine ili kupunguza wingi wa seli za vimelea na kuenea kwenda kwa mbu.

Njia

Katika utafiti huu wenye afua nne, uliofumbwa upande mmoja, awamu ya pili, utafiti wa jaribio la nasibu washiriki waliandikishwa katika Kitengo cha Tafiti za Afya cha Kituo cha Mafunzo na Tafiti za Malaria katika Chuo Kikuu cha Bamako, Mali. Washiriki waliofikia vigezo walikua na umri wa miaka 12 mpaka 50 wasio wagonjwa lakini wenye maambukizi ya P falciparum na seli za vimelea vilivyogunduliwa na darubini, wenye uzito wa mwili wa kilo 80 au pungufu ya hapo, na hawakua na viashiria vya kitabibu vya malaria vinavyotokana na homa. Washiriki walipangwa kiholela (1:1:1:1) kwenye tiba ya kiwango yenye dihydroartemisinin–piperaquine, au dihydroartemisinin–piperaquine pamoja na dozi ya mara moja ya tafenoquine (ya maji) wakati wa dozi ya mwisho ya 0·42 mg/kg, 0·83 mg/kg, au 1·66 mg/kg. Mpangilio wa kiholela ulifanyika kwa orodha iliyotengenezwa kwa kompyuta kwa kupanga uholela na kufungwa kwa muhuri kwenye bahasha zisizoonesha. Dihydroartemisinin–piperaquine ilitolewa kwa vidonge vya kumezwa kwa siku 3 (siku 0, 1, na 2) kulingana na maelekezo ya mtengenezaji. Dozi moja ya tafenoquine ilitolewa ikiwa ni ya kumezwa ya maji siku ya kuanza (siku 0) sambamba na dozi ya kwanza ya dihydroartemisinin–piperaquine. Utolewaji wa dozi ya tafenoquine ulizingatia uzito wa mwili ili kusanifisha tofauti ya uwezo na madhara. Matokeo ya msingi, yaliyotathminiwa kulingana na idadi ya watu wa utafiti ilikuwa wastani wa asilimia ya kiwango cha mabadiliko ya uambukizo kwa mbu siku 7 baada ya kupata tiba ikilinganishwa na mwanzoni. Usalama wa matokeo ya mwisho ulijumuisha kurudia na kutokea kwa matukio ya madhara. Kutembelewa kwa ufuatiliaji wa mwisho ilikuwa tarehe 23 mwezi wa Decemba mwaka 2021; utafiti wa majaribio uliandikishwa ClinicalTrials.gov, NCT04609098.

Matokeo

Kuanzia October 29 mpaka Novemba 25, mwaka 2020, jumla ya watu 1091 walichunguzwa kwa vigezo, watu 80 kati yao waliandikishwa na kupangwa kiholela (watu 20 kwa kundi moja la tiba). Kabla ya tiba, watu 53 (66%) walikua wanaweza kuambukiza mbu, wastani wa uambukizo ukiwa 12.50% ya mbu (IQR 3·64–35·00). Kiwango cha punguzo la uambukizo wa mbu kwa kundi siku ya 7 kilikuwa 79·95% (IQR 57·15–100; p=0·0005 kwa tofauti ya kuanzia mwanzoni) kutokana na dihydroartemisinin–piperaquine tu, 100% (98·36–100; p=0·0005) kutokana na dihydroartemisinin–piperaquine pamoja na tafenoquine 0·83 mg/kg, na 100% (100–100; p=0·0001) kutokana na dihydroartemisinin–piperaquine pamoja na tafenoquine 1·66 mg/kg. Washiriki 55 (69%) kati ya 80 walikua na jumla ya matukio ya madhara 94 ndani ya kipindi cha utafiti wa majaribio; 86 (92%) ya matukio ya madhara yaliainishwa kama madogo, saba (7%) kama ya wastani na moja (1%) kama makali. Matukio ya madhara ya mara kwa mara yaliyohusiana na tiba yalikuwa madogo au ya wastani kwa maumivu ya kichwa ambayo yalitokea kwa washiriki 15 (19%) ambapo (dihydroartemisinin–piperaquine idadi=2; dihydroartemisinin– piperaquine pamoja na tafenoquine 0·42 mg/kg idadi=6; dihydroartemisinin–piperaquine pamoja na tafenoquine 0·83 mg/kg idadi=3; na dihydroartemisinin–piperaquine pamoja na tafenoquine 1·66 mg/kg idadi=4). Hakuna matukio ya madhara makali yaliyotokea. Hakukuwa na tofauti za muhimu wakati wa kutokea kwa matukio yote

ya madhara (p=0.73) au matukio ya madhara yaliyohusiana na tiba (p=0.62) yalionekana kati ya makundi ya tiba.

Tafsiri

Tafenoquine ilivumiliwa vizuri katika dozi zote na iliongeza kasi ya P falciparum kumaliza seli za vimelea. Dozi zote za tafenoquine zilionesha kuboresha punguzo la uambukizo katika siku ya 7 ikilinganishwa na dihydroartemisinin–piperaquine pekee. Takwimu hizi zitasaidia fani hii ili kufanyika tafiti zaidi kwenye tafenoquine ambayo ni dawa ya kuzuia kusambaa kama nyongeza ya dawa za kutibu malaria zilizopo.
