# Supplementary material for: Single low-dose tafenoquine combined with dihydroartemisinin–piperaquine to reduce Plasmodium falciparum transmission in Ouelessebougou, Mali: a phase 2, single-blind, randomised clinical trial
Source: Lancet Microbe. 2022 May;3(5):e336–47. doi: 10.1016/S2666-5247(21)00356-6 (PMC9042793; doi:10.1016/S2666-5247(21)00356-6)
Supplement: Supplementary appendix 5 [file mmc5.pdf]

THE LANCET
Microbe

Supplementary appendix 5

This appendix formed part of the original submission and has been peer reviewed. We post it as supplied by the authors.

Supplement to: Stone W, Mahamar A, Smit MJ, et al. Single low-dose tafenoquine combined with dihydroartemisinin–piperaquine to reduce *Plasmodium falciparum* transmission in Ouelessebougou, Mali: a phase 2, single-blind, randomised clinical trial. *Lancet Microbe* 2022; published online March 23. https://doi.org/10.1016/S2666-5247(21)00356-6.

Appendix

Supplementary information 1. Antimalarial treatment dosing 2
Supplementary figure 1. Schematic representation of sample collection and analysis pipeline 4
Supplementary table 1. Primer sequences and qPCR conditions for PfMGET CCp4 assay 5
Supplementary table 2. Infectivity to mosquitoes 6
Supplementary table 3. Gametocyte circulation time and area under the curve 7
Supplementary table 4A. Total gametocyte density, prevalence and sex ratio 8
Supplementary table 5. Female (CCP4) and male (PfMGET) gametocyte density and prevalence 9
Supplementary figure 2. Proportion of gametocytes that were male 10
Supplementary table 6. Haemoglobin density 11
Supplementary table 7. Methaemoglobin concentration 12
Supplementary figure 3. Haemoglobin and Methaemoglobin 13
Supplementary table 8. Prevalence of adverse events 14
Supplementary table 9. Frequency of all adverse events 15
Supplementary table 10. Biochemistry 16
References 17

Supplementary information 1. Antimalarial treatment dosing

A. *Dihydroartemisinin-Piperaquine (DP)*

Participants in the DP or DP-PQ arm were treated with standard doses of DP from day 0-2. DP treatment Tablets containing 160/320 mg piperaquine w. 20/40 mg dihydroartemisinin tablets (Eurartesim, Sigma Tau) were administered according to weight as per manufacturer guidelines shown below:

Body weight (kg)	Total daily dose (mg)<br>(1x/day for 3 days)		Tablet strength and number of tablets per dose
	Piperaquine	DHA	
5 to <7	80	10	½ x 160mg / 20mg
7 to <13	160	20	1 x 160mg / 20mg
13 to <24	320	40	1 x 320mg / 40mg
24 to <36	640	80	2 x 320mg / 40mg
36 to <75	960	120	3 x 320mg / 40mg
75 to 80	1,280	160	4 x 320mg / 40mg
>80	Not eligible		

B. *Tafenoquine*

A single dose of TQ was given on day 0 in parallel with the first dose of DP, at one of three doses: 1.66mg/kg, 0.83mg/kg, or 0.42mg/kg. 1.66mg/kg is equivalent to a 100mg single dose in a 60kg adult. The maximum dose chosen for the current study was based on the reported safety profile of TQ doses ≤300mg, which is similar to that of standard PQ dosing (15mg daily for 14 days) in adult G6PD heterozygous adult individuals (14).

100mg Tafenoquine tablets were available for this study, and were prepared into a 1mg/mL solution in water for weight-based dosing in 5 kg bands as follows:

Arm 1. 1.66mg/kg TQ

Weight min	Weight max	TQ 1mg/mL total (mL)	Water (mL)	Masking solution (mL)
30	35	54.0	136.1	10
35.01	40	62.3	127.7	10
40.01	45	70.6	119.4	10
45.01	50	78.9	111.1	10
50.01	55	87.2	102.8	10
55.01	60	95.5	94.5	10
60.01	65	103.8	86.2	10
65.01	70	112.1	77.9	10
70.01	75	120.4	69.6	10
75.01	80	128.7	61.3	10

Arm 2. 0.83mg/kg TQ

Weight min	Weight max	TQ 1mg/mL total (mL)	Water (mL)	Masking solution (mL)
30	35	27.0	163.0	10
35.01	40	31.1	158.9	10
40.01	45	35.3	154.7	10
45.01	50	39.4	150.6	10
50.01	55	43.6	146.4	10
55.01	60	47.7	142.3	10
60.01	65	51.9	138.1	10
65.01	70	56.0	134.0	10

70.01	75	60.2	129.8	10
75.01	80	64.3	125.7	10

Arm 3. 0.415mg/kg TQ

Weight min	Weight max	TQ 1mg/mL total (mL)	Water (mL)	Masking solution (mL)
30	35	13.5	176.5	10
35.01	40	15.6	174.4	10
40.01	45	17.6	172.4	10
45.01	50	19.7	170.3	10
50.01	55	21.8	168.2	10
55.01	60	23.9	166.1	10
60.01	65	25.9	164.1	10
65.01	70	28.0	162.0	10
70.01	75	30.1	159.9	10
75.01	80	32.2	157.8	10

Supplementary figure 1. Schematic representation of sample collection and analysis pipeline

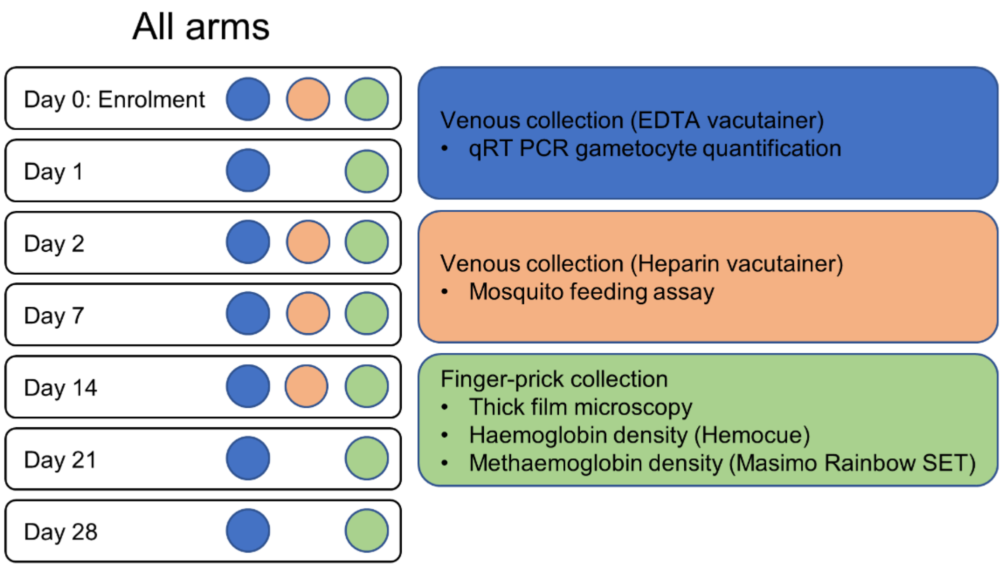

Supplementary table 1. Primer sequences and qPCR conditions for PfMGET CCp4 assay

PfMGET Primer/Probe Sequences

Primers	Sequence
Primer-FW (5'-3')	CGGTCCAAATATAAAAATCCTG
Primer-RV (5'-3')	TGTG TAACG TATG ATTCATTTTC
Probe (5'-3')	FAM-CAGCTCCAG CATTAAAACAC-BHQ1

CCp4 Primer/Probe Sequences

Primers	Sequence
Primer-FW (5'-3')	CACATGAATATGAGAATAAAATTG
Primer-RV (5'-3')	TAGGCGAACATGTGGAAAG
Probe (5'-3')	TexasRed-AGCAACAACGGTATGTGCCTTAAAACG-BHQ2

Male and female gametocyte quantification was performed as described previously, using a multiplex RT-qPCR assay (1). Assays were run using commercial RT-qPCR mixes (Luna® Universal Probe One-Step RT-qPCR Kit, New England Biolabs, Ipswich, MA, USA). FW = Forward primer. RV = Reverse primer.

Supplementary table 2. Infectivity to mosquitoes

Day of follow-up	Treatment arm	Infectious individuals* % (n/N)	p-value¥	p-value†	Mosquito infection rate** Median % (IQR)	p-value¥	p-value†	Oocyst density Median (IQR)***	p-value¥	p-value†
Day 0	Overall	66 (53/80)	-	-	12.5 (3.64-35)	-	-	1.5 (1-2.33)	-	-
	DP	60 (12/20)	*ref*	*ref*	12.5 (2.44-35.24)	*ref*	*ref*	1.57 (1-3.16)	*ref*	*ref*
	DP-TQ 0.42mg/kg	65 (13/20)	*ref*	0.50	12.96 (6.67-28.79)	*ref*	0.57	1.4 (1-1.58)	*ref*	0.63
	DP-TQ 0.83mg/kg	70 (14/20)	*ref*	0.37	13.39 (3.03-56.52)	*ref*	0.64	1.81 (1-3.15)	*ref*	0.83
	DP-TQ 1.66mg/kg	70 (14/20)	*ref*	0.37	11.35 (6.82-29.17)	*ref*	0.75	1.51 (1-2.21)	*ref*	0.54
Day 2	DP	45 (9/20)	0.26	*ref*	8.63 (0-31.92)	0.42	*ref*	1.66 (1.2-2.35)	0.22	*ref*
	DP-TQ 0.42mg/kg	58 (11/19)	0.45	0.31	9.39 (4.09-19.71)	0.016	0.7	1.36 (1-5.2)	0.094	0.74
	DP-TQ 0.83mg/kg	75 (15/20)	0.50	0.053	19.77 (7.35-43.08)	0.9	0.19	2 (1.6-3.87)	0.12	0.53
	DP-TQ 1.66mg/kg	65 (13/20)	0.50	0.17	7.59 (2.38-26.79)	0.53	0.85	1.33 (1-3.42)	0.46	0.55
Day 7	DP	50 (10/20)	0.38	*ref*	2.36 (0-10.39)	0.0005	*ref*	1.21 (1-1.92)	0.25	*ref*
	DP-TQ 0.42mg/kg	16 (3/19)	0.0022	0.026	0 (0-1.52)	0.0005	0.056	1 (1-1.33)	0.25	0.49
	DP-TQ 0.83mg/kg	10 (2/20)	0.0001	0.0069	0 (0-0)	0.0001	0.0034	1 (1-1)	0.5	0.44
	DP-TQ 1.66mg/kg	0 (0/19)	<0.0001	0.0003	0 (0-0)	0.0001	0.0006	*nc*	*nc*	*nc*
Day 14	DP	16 (3/19)	0.0054	*ref*	0 (0-1.06)	0.0005	*ref*	1 (1-1)	0.25	*ref*
	DP-TQ 0.42mg/kg	0 (0/19)	<0.0001	0.12	0 (0-0)	0.0005	0.22	*nc*	*nc*	*nc*
	DP-TQ 0.83mg/kg	0 (0/19)	<0.0001	0.11	0 (0-0)	0.0001	0.17	*nc*	*nc*	*nc*
	DP-TQ 1.66mg/kg	0 (0/17)	<0.0001	0.136	0 (0-0)	0.0005	0.22	*nc*	*nc*	*nc*

Percentage of infectious individuals. *Individuals were classed as infectious if direct membrane feeding assays (DMFA) resulted in at least one mosquito with any number of oocysts. Mosquito infection measures (percent infection and oocyst density) are presented for all participants who were infectious at baseline, and oocyst densities are from all infected mosquitoes **Mosquito infection rate = Median percentage of mosquitoes infected by each participant, where for each participant mosquito infection rate the number of mosquitoes infected as a percentage of all mosquitoes surviving to dissection. ***The average oocyst density for each participant was calculated as the mean number of oocysts in infected mosquitoes (i.e., with at least one oocyst). The value presented in the table is the median of all individuals' average oocyst intensities (a composite figure of all oocysts/all infected mosquitoes is not statistically valid). *nc* = not calculable, no positive observations. - = not tested. P-value¥ = Within group comparison. P-value† = Between group comparison (TQ groups with DP reference group). *ref* = reference group.

Supplementary table 3. Gametocyte circulation time and area under the curve

	Treatment group	Total gametocytes (CCP4 & PfMGET)	p-value	Female gametocytes (CCP4)	p-value	Male gametocytes (PfMGET)	p-value	p-value♂♀
Circulation time Days (95% CI)	DP	8.26 (6.96-9.55)	*ref*	8.02 (6.64-9.40)	*ref*	7.66 (6.49-8.84)	*ref*	0.57
	DP-TQ (0.42mg/kg)	4.43 (4.03-4.82)	<0.0001*	5.05 (4.48-5.63)	0.0002*	3.28 (3.02-3.54)	<0.0001*	<0.0001
	DP-TQ (0.83mg/kg)	3.61 (3.36-3.87)	<0.0001*	4.01 (3.65-4.36)	<0.0001*	2.85 (2.65-3.05)	<0.0001*	<0.0001
	DP-TQ (1.66mg/kg)	2.67 (2.48-2.86)	<0.0001*	2.79 (2.55-3.03)	<0.0001*	2.32 (2.13-2.51)	<0.0001*	0.012
Circulation time (dose response)		0.48 (0.30-0.65)	<0.0001***	1.19 (0.86-1.52)	<0.0001***	0.91 (0.68-1.15)	<0.0001*	-
AUC Median (IQR) gametocytes per uL/day	DP	11.87 (5.88-50.97)	*ref*	5.14 (3.02-23.95)	*ref*	6.38 (3.70-22.99)	*ref*	0.053
	DP-TQ (0.42mg/kg)	6.88 (4.09-18.74)	0.046*	4.66 (1.95-12.81)	0.52*	4.00 (2.25-12.33)	0.054*	0.42
	DP-TQ (0.83mg/kg)	13.47 (4.18-70.30)	0.0085*	7.38 (2.03-37.84)	0.066*	7.48 (2.25-42.77)	0.037*	0.21
	DP-TQ (1.66mg/kg)	7.47 (3.81-39.73)	<0.0001*	3.71 (2.07-18.05)	<0.0001*	5.31 (2.20-22.70)	0.0075*	0.017

Gametocyte circulation time was calculated using a deterministic compartmental model (4), and is presented as the model estimate (mean days) with 95% CI. Area under the curve (AUC) of gametocyte density per participant over time was calculated using the linear trapezoid method (5), and is presented as the median and IQR of individual AUC values by treatment arm. P-values (*) are for differences in the t-statistic between TQ treatment groups and the DP reference group (*), for between sexes within treatment groups (♂♀). or for the dose-response of circulation with doubling doses of TQ (**) or *ref* = reference group.

Supplementary table 4A. Total gametocyte density, prevalence and sex ratio

	Total gametocytes (CCP4 & PfMGET)						
Day of follow-up	Treatment arm	Median gametocytes/µL (IQR)	p-value	Prevalence % (n/N)	p-value	Proportion male Median p (IQR)	p-value
Day 0	*Overall*	41.52 (12.5-95.9)	-	100 (80/80)	-	0.51 (0.42-0.62)	-
	DP	29.46 (12.34-67.76)	*ref*	100 (20/20)	*ref*	0.57 (0.42-0.68)	*ref*
	DP-TQ (0.42mg/kg)	43.73 (18.5-244.99)	0.57	100 (20/20)	*nc*	0.49 (0.41-0.61)	0.19
	DP-TQ (0.83mg/kg)	50.12 (17.75-201.82)	0.28	100 (20/20)	*nc*	0.51 (0.47-0.6)	0.30
	DP-TQ (1.66mg/kg)	24.12 (8.36-66.11)	0.35	100 (20/20)	*nc*	0.5 (0.41-0.59)	0.25
Day 1	DP	22.5 (9.39-50.82)	*ref*	100 (18/18)	*ref*	0.56 (0.46-0.65)	*ref*
	DP-TQ (0.42mg/kg)	43.21 (16.11-152.81)	1.0	100 (19/19)	*nc*	0.47 (0.36-0.58)	0.12
	DP-TQ (0.83mg/kg)	45.14 (18.78-128.51)	0.13	100 (20/20)	*nc*	0.5 (0.41-0.56)	0.13
	DP-TQ (1.66mg/kg)	24.65 (8.79-92.03)	*0.19*	100 (17/17)	*nc*	0.5 (0.37-0.57)	0.16
Day 2	DP	23.46 (10.37-69.15)	*ref*	100 (20/20)	*ref*	0.57 (0.49-0.73)	*ref*
	DP-TQ (0.42mg/kg)	45.24 (14.68-135)	0.58	100 (19/19)	*nc*	0.51 (0.37-0.65)	0.10
	DP-TQ (0.83mg/kg)	34.49 (15.39-122.51)	0.32	100 (20/20)	*nc*	0.54 (0.46-0.59)	0.18
	DP-TQ (1.66mg/kg)	11.56 (6.26-43.51)	0.43	100 (20/20)	*nc*	0.52 (0.47-0.62)	0.24
Day 7	DP	9.97 (6.13-29.33)	*ref*	100 (19/19)	*ref*	0.54 (0.5-0.67)	*ref*
	DP-TQ (0.42mg/kg)	16.07 (7.33-53.14)	0.77	100 (19/19)	*nc*	0.54 (0.38-0.71)	0.70
	DP-TQ (0.83mg/kg)	5.26 (1.5-25.14)	0.96	100 (19/19)	*nc*	0.61 (0.53-0.8)	0.25
	DP-TQ (1.66mg/kg)	8.79 (3.97-40.23)	0.049	100 (19/19)	*nc*	0.9 (0.81-0.96)	0.0002
Day 14	DP	2.21 (0.93-3.88)	*ref*	100 (19/19)	*ref*	0.57 (0.39-0.67)	*ref*
	DP-TQ (0.42mg/kg)	0.86 (0.32-3.28)	0.0003	89 (17/19)	0.24	0.18 (0.05-0.48)	0.0004
	DP-TQ (0.83mg/kg)	0.18 (0-0.38)	<0.0001	85 (17/20)	0.13	0.22 (0.04-0.45)	0.0048
	DP-TQ (1.66mg/kg)	3.67 (0.97-7.58)	<0.0001	63 (10/16)	0.0049	0.39 (0.13-0.85)	0.50
Day 21	DP	0.3 (0.17-1.92)	*ref*	100 (20/20)	*ref*	0.51 (0.35-0.7)	*ref*
	DP-TQ (0.42mg/kg)	0.23 (0.07-0.47)	0.034	79 (15/19)	0.047	0.05 (0-0.15)	<0.0001
	DP-TQ (0.83mg/kg)	0 (0-0.07)	0.0005	85 (17/20)	0.115	0.13 (0.08-0.26)	<0.0001
	DP-TQ (1.66mg/kg)	1.41 (0.26-2.86)	0.0041	39 (7/18)	<0.0001	0.02 (0.02-0.02)	0.10
Day 28	DP	0.06 (0-0.46)	*ref*	90 (18/20)	*ref*	0.43 (0.28-0.72)	*ref*
	DP-TQ (0.42mg/kg)	0.03 (0-0.15)	0.18	67 (12/18)	0.086	0.04 (0-0.08)	0.0043
	DP-TQ (0.83mg/kg)	0 (0-0)	0.019	65 (13/20)	0.064	0.06 (0.01-0.18)	0.0050
	DP-TQ (1.66mg/kg)	41.52 (12.5-95.9)	0.11	17 (3/18)	<0.0001	0 (0-0)	0.12

P-values are for differences between TQ groups and the reference (DP) group. Density was compared using regression analyses of log10 transformed density values, with adjustment for baseline densities. Prevalence was compared with one sided Fishers exact tests. For males and females, Proportion male is given for participants/time-points with total gametocyte densities of 0.2/µL and over, as described previously (3). For the calculation of gametocyte prevalence, samples were classified as negative for a particular gametocyte sex if the estimated density of in gametocytes of that sex was less than 0·01/µL (i.e. one gametocyte per 100 µL of blood sample). *nc* = not calculable, no observations/no observations over the threshold density for analysis. - = not tested. *ref* = reference group.

4B. Gametocyte infectivity

Day of follow-up	Treatment arm	Log odds ratio (95% CI)	*p-value*
Day 2	DP	1 *(base)*	*ref*
	DP-TQ (0.42mg/kg)	0.96 (0.67-1.36)	0.800
	DP-TQ (0.83mg/kg)	1.25 (0.92-1.68)	0.152
	DP-TQ (1.66mg/kg)	0.82 (0.61-1.10)	0.193
Day 7	DP	1 *(base)*	*ref*
	DP-TQ (0.42mg/kg)	0.33 (0.14-0.79)	0.014
	DP-TQ (0.83mg/kg)	0.031 (0.0041-0.24)	0.001
	DP-TQ (1.66mg/kg)	*nc*	*nc*

Log odds ratios are for the change in mosquito infection rate in the TQ arms compared to the reference (DP) arm, with adjustment for female and male gametocyte densities. *nc* = not calculable, no observations (too few infected mosquitoes for convergence). *ref* = reference group.

Supplementary table 5. Female (CCP4) and male (PfMGET) gametocyte density and prevalence

		Female gametocytes (CCP4)				Male gametocytes (PfMGET)			
Day of follow-up	Treatment arm	Median/µL (IQR)	p-value	Prevalence % (n/N)	p-value	Median/µL (IQR)	p-value	Prevalence % (n/N)	p-value
Day 0	*Overall*	20.21 (7.64-74.39)	-	100 (80/80)	-	21.43 (6.8-67.1)	-	100 (80/80)	-
	DP	17.45 (6.34-43.18)	*ref*	100 (20/20)	*ref*	17.72 (6.3-48.29)	*ref*	100 (20/20)	*ref*
	DP-TQ (0.42mg/kg)	9.1 (6.19-33.5)	0.64	100 (20/20)	*nc*	15.26 (5.8-36.46)	0.59	100 (20/20)	*nc*
	DP-TQ (0.83mg/kg)	22.3 (7.46-91.53)	0.16	100 (20/20)	*nc*	24.26 (7.34-159.53)	0.61	100 (20/20)	*nc*
	DP-TQ (1.66mg/kg)	23.52 (12.25-85.74)	0.24	100 (20/20)	*nc*	25.52 (9.06-107.83)	0.49	100 (20/20)	*nc*
Day 1	DP	10 (2.95-24.32)	*ref*	100 (18/18)	*ref*	9.03 (3.95-37.05)	*ref*	100 (18/18)	*ref*
	DP-TQ (0.42mg/kg)	11.95 (4.09-31.83)	0.86	100 (19/19)	*nc*	10.55 (4.46-24.59)	0.96	100 (19/19)	*nc*
	DP-TQ (0.83mg/kg)	23.81 (7.13-63.87)	0.053	100 (20/20)	*nc*	21.61 (6.39-89.7)	0.45	100 (20/20)	*nc*
	DP-TQ (1.66mg/kg)	24.93 (9.13-36.59)	0.14	100 (17/17)	*nc*	19.11 (7.8-68.26)	0.30	100 (17/17)	*nc*
Day 2	DP	8.31 (2.97-38.49)	*ref*	100 (20/20)	*ref*	12.55 (5.25-52.75)	*ref*	100 (20/20)	*ref*
	DP-TQ (0.42mg/kg)	12.64 (4.01-41.49)	0.74	100 (19/19)	*nc*	10.14 (6.16-29.02)	0.55	100 (19/19)	*nc*
	DP-TQ (0.83mg/kg)	21.09 (5.08-70)	0.18	100 (20/20)	*nc*	17.99 (5.83-76.91)	0.77	100 (20/20)	*nc*
	DP-TQ (1.66mg/kg)	15.26 (7.07-47.22)	0.37	100 (20/20)	*nc*	16.92 (8.32-61.87)	0.50	100 (20/20)	*nc*
Day 7	DP	5.23 (2.19-21.35)	*ref*	95 (18/19)	*ref*	7.48 (2.48-17.88)	*ref*	100 (19/19)	*ref*
	DP-TQ (0.42mg/kg)	4.37 (2.09-17.46)	0.46	95 (18/19)	0.76	6.49 (2.72-21.19)	0.90	100 (19/19)	*nc*
	DP-TQ (0.83mg/kg)	4.7 (0.83-14.22)	0.37	100 (19/19)	0.50	7.37 (3.85-29.49)	0.86	100 (19/19)	*nc*
	DP-TQ (1.66mg/kg)	0.52 (0.1-1.14)	0.0002	84 (16/19)	0.30	4.74 (1.43-24.2)	0.35	100 (19/19)	*nc*
Day 14	DP	3.99 (1.57-16.05)	*ref*	95 (18/19)	*ref*	4.08 (1.97-20.93)	*ref*	100 (19/19)	*ref*
	DP-TQ (0.42mg/kg)	1.39 (0.68-2.64)	0.0059	84 (16/19)	0.30	0.3 (0.06-1.51)	<0.0001	84 (16/19)	0.12
	DP-TQ (0.83mg/kg)	0.7 (0.21-1.66)	<0.0001	85 (17/20)	0.32	0.23 (0-0.62)	<0.0001	70 (14/20)	0.012
	DP-TQ (1.66mg/kg)	0.05 (0-0.22)	<0.0001	63 (10/16)	0.024	0.01 (0-0.22)	0.0002	50 (8/16)	0.0005
Day 21	DP	1.85 (0.21-5.23)	*ref*	95 (19/20)	*ref*	1.76 (0.45-2.83)	*ref*	100 (20/20)	*ref*
	DP-TQ (0.42mg/kg)	0.28 (0.17-1.89)	0.25	79 (15/19)	0.16	0.03 (0-0.13)	0.0043	53 (10/19)	0.0004
	DP-TQ (0.83mg/kg)	0.22 (0.05-0.33)	0.010	80 (16/20)	0.17	0.03 (0-0.09)	0.0014	60 (12/20)	0.0016
	DP-TQ (1.66mg/kg)	0 (0-0.06)	0.030	33 (6/18)	<0.0001	0 (0-0)	0.065	17 (3/18)	<0.0001
Day 28	DP	0.6 (0.14-1.48)	*ref*	85 (17/20)	*ref*	0.48 (0.11-1.33)	*ref*	90 (18/20)	*ref*
	DP-TQ (0.42mg/kg)	0.06 (0-0.24)	0.82	67 (12/18)	0.17	0 (0-0)	0.36	22 (4/18)	<0.0001
	DP-TQ (0.83mg/kg)	0.03 (0-0.15)	0.12	65 (13/20)	0.14	0 (0-0)	0.36	20 (4/20)	<0.0001
	DP-TQ (1.66mg/kg)	0 (0-0)	0.24	17 (3/18)	<0.0001	0 (0-0)	0.64	6 (1/18)	<0.0001

P-values are for differences between TQ groups and the reference (DP) group. Density was compared using regression analyses of log10 transformed density values, with adjustment for baseline densities. Prevalence was compared with one sided Fishers exact tests. For the calculation of gametocyte prevalence, samples were classified as negative for a particular gametocyte sex if the estimated density of in gametocytes of that sex was less than 0·01 gametocytes per µL (i.e. one gametocyte per 100 µL of blood sample). - = not tested. *ref* = reference group.

Supplementary figure 2. Proportion of gametocytes that were male

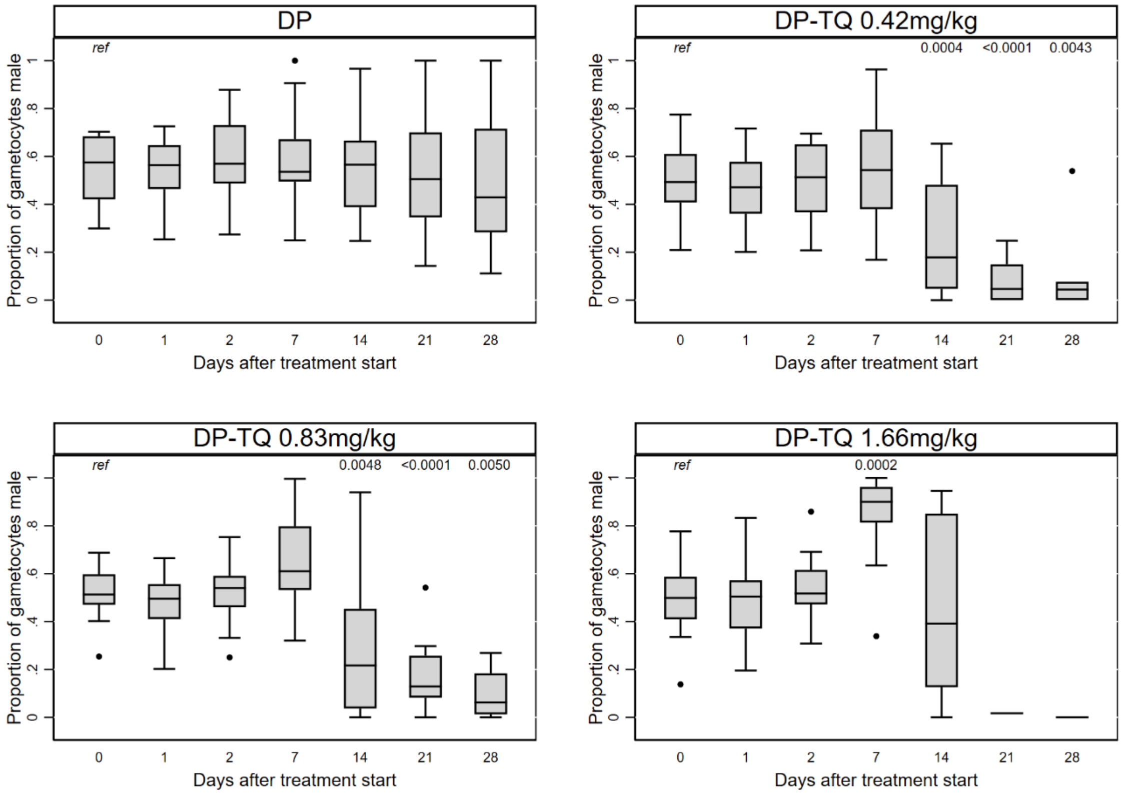

The proportion of gametocytes that were male was calculated for all values with total gametocyte densities of 0.2/μL and over, as described previously (3). P-values (<0.05) for differences between TQ treatment groups and the reference (DP) group were calculated using Wilcoxon rank sum tests.

Supplementary table 6. Haemoglobin density

Day of follow-up	Treatment arm	Mean g/dL (range)	p-value¥	p-value†	Percent change from day 0			
					Mean (Lower/upper 95% CI)	Range	p-value¥	p-value†
Day 0	*Overall*	12.64 (10.2-15.8)	-	-	-	-	-	-
	DP	12.97 (10.9-15.7)	*ref*	*ref*	-	-	-	-
	DP-TQ (0.42mg/kg)	12.13 (10.2-15.8)	*ref*	0.040	-	-	-	-
	DP-TQ (0.83mg/kg)	12.69 (11.1-14.8)	*ref*	0.49	-	-	-	-
	DP-TQ (1.66mg/kg)	12.8 (10.8-15)	*ref*	0.67	-	-	-	-
Day 1	DP	12.87 (10.7-15.5)	0.38	*ref*	-0.78 (-2.6/1.03)	-9.32/7.09	0.38	*ref*
	DP-TQ (0.42mg/kg)	12.07 (9.9-14.4)	0.78	0.81	-0.18 (-4.09/3.73)	-13.95/14.42	0.92	0.77
	DP-TQ (0.83mg/kg)	12.59 (10.7-15.7)	0.43	*0.91*	-0.72 (-2.59/1.16)	-6.96/8.04	0.43	0.96
	DP-TQ (1.66mg/kg)	12.65 (10.8-15.3)	0.43	0.78	-1.07 (-3.9/1.76)	-14.29/11.63	0.44	0.86
Day 2	DP	12.71 (11.3-15.8)	0.049	*ref*	-1.87 (-3.89/0.16)	-9.22/7.34	0.068	*ref*
	DP-TQ (0.42mg/kg)	11.84 (10-15.6)	0.20	0.61	-2.22 (-5.96/1.52)	-12.4/16.42	0.23	0.86
	DP-TQ (0.83mg/kg)	12.45 (10.6-14.8)	0.033	0.99	-1.95 (-3.69/-0.21)	-7.83/9.6	0.030	0.95
	DP-TQ (1.66mg/kg)	12.56 (10.5-14.8)	0.19	0.97	-1.73 (-4.5/1.04)	-13.53/7.03	0.21	0.93
Day 7	DP	12.54 (11-16.4)	0.0003	*ref*	-3.26 (-4.73/-1.79)	-6.78/4.46	0.0002	*ref*
	DP-TQ (0.42mg/kg)	12.02 (10.1-14.8)	0.51	0.23	-0.56 (-3.71/2.58)	-8.53/14.42	0.71	0.11
	DP-TQ (0.83mg/kg)	12.48 (10.6-14.9)	0.010	0.29	-1.66 (-2.87/-0.46)	-5.04/3.48	0.0094	0.087
	DP-TQ (1.66mg/kg)	12.45 (10.1-14.8)	0.078	0.57	-2.32 (-4.88/0.25)	-15.04/6.47	0.074	0.50
Day 14	DP	12.79 (11.5-15.6)	0.29	*ref*	-1 (-3.45/1.44)	-10.14/12.84	0.40	*ref*
	DP-TQ (0.42mg/kg)	12.02 (10.2-14.7)	0.58	0.44	-0.49 (-3.94/2.97)	-16.46/10.78	0.77	0.80
	DP-TQ (0.83mg/kg)	12.69 (11.1-15)	0.96	0.67	0.12 (-1.64/1.89)	-7.43/5.17	0.89	0.44
	DP-TQ (1.66mg/kg)	12.64 (10.8-14.7)	0.24	0.60	-1.85 (-5.38/1.67)	-18.05/11.61	0.28	0.67
Day 21	DP	12.84 (10.6-15.4)	0.66	*ref*	-0.63 (-5.23/3.96)	-24.82/18.55	0.78	*ref*
	DP-TQ (0.42mg/kg)	12.34 (10.3-14.8)	0.48	0.98	2.36 (-2.35/7.07)	-14.56/16.67	0.31	0.35
	DP-TQ (0.83mg/kg)	12.86 (11.3-15)	0.17	0.56	1.56 (-0.44/3.55)	-4.32/10.43	0.12	0.37
	DP-TQ (1.66mg/kg)	12.82 (9.9-15)	0.67	0.99	-0.73 (-5.55/4.09)	-25.56/16.28	0.75	0.98
Day 28	DP	12.94 (10.9-16.1)	0.89	*ref*	0.03 (-2.81/2.88)	-11.49/11.02	0.98	*ref*
	DP-TQ (0.42mg/kg)	12.31 (10.7-13.9)	0.038	0.83	3.5 (0.36/6.65)	-3.88/19.42	0.03	0.093
	DP-TQ (0.83mg/kg)	13.01 (11.7-14.6)	0.045	0.28	2.89 (0.31/5.48)	-6.34/14.41	0.03	0.13
	DP-TQ (1.66mg/kg)	13.02 (10.3-15.5)	0.79	0.70	0.85 (-3.57/5.26)	-22.56/15.04	0.69	0.74

Haemoglobin density and percent change in haemoglobin density (relative to baseline) were compared within treatment arms (p-value¥) using paired t-tests (with day 0 as reference for percent change) and between treatment arms (p-value†) using linear regression (for density, adjusted for baseline Hb density) or two-way t-tests (for percent reduction).

Supplementary table 7. Methaemoglobin concentration

Day of follow-up	Treatment arm	Mean Met Hb % (range)	p-value¥	p-value†	Percent change from day 0 Mean % (Lower/upper 95% CI)	p-value¥	p-value†
Day 0	*Overall*	1.53 (0.9-2.7)	-	-	-	-	-
	DP	1.53 (0.9-2.5)	*ref*	*ref*	-	-	-
	DP-TQ (0.42mg/kg)	1.51 (0.9-2.7)	*ref*	0.87	-	-	-
	DP-TQ (0.83mg/kg)	1.45 (1-2.3)	*ref*	0.52	-	-	-
	DP-TQ (1.66mg/kg)	1.63 (1.1-2.5)	*ref*	0.37	-	-	-
Day 1	DP	1.49 (0.9-2.2)	0.67	*ref*	0.76 (-12.24/13.76)	0.91	*ref*
	DP-TQ (0.42mg/kg)	1.63 (1.1-3)	0.12	0.17	11.04 (-1.53/23.61)	0.082	0.24
	DP-TQ (0.83mg/kg)	1.52 (1.1-2.2)	0.28	*0.45*	7.87 (-1.39/17.13)	0.091	0.36
	DP-TQ (1.66mg/kg)	1.78 (0.9-3.3)	0.15	0.046	9.47 (-2.69/21.63)	0.12	0.31
Day 2	DP	1.54 (1.1-2)	0.92	*ref*	4.77 (-8.45/17.99)	0.46	*ref*
	DP-TQ (0.42mg/kg)	1.59 (0.9-2.5)	0.31	0.55	9.89 (-3.63/23.4)	0.14	0.57
	DP-TQ (0.83mg/kg)	1.59 (1.1-2.5)	0.12	0.39	13.19 (-1.06/27.44)	0.068	0.37
	DP-TQ (1.66mg/kg)	1.77 (1.2-2.6)	0.10	0.048	11.53 (-1.6/24.66)	0.082	0.45
Day 7	DP	1.61 (1.3-2.3)	0.31	*ref*	8.46 (-2.3/19.23)	0.12	*ref*
	DP-TQ (0.42mg/kg)	1.64 (1-2.7)	0.15	0.64	13.09 (-1.54/27.72)	0.076	0.59
	DP-TQ (0.83mg/kg)	1.65 (1.2-2.1)	0.0074	0.38	17.45 (6.41/28.49)	0.0037	0.23
	DP-TQ (1.66mg/kg)	1.93 (1.3-3.2)	0.0006	0.004	20.62 (9.39/31.84)	0.0011	0.11
Day 14	DP	1.57 (1.2-2.2)	0.58	*ref*	7.3 (-4.91/19.5)	0.23	*ref*
	DP-TQ (0.42mg/kg)	1.74 (1.1-3)	0.052	0.15	21.16 (0.48/41.85)	0.045	0.23
	DP-TQ (0.83mg/kg)	1.63 (1.2-2.2)	0.041	0.43	16.78 (3.43/30.12)	0.016	0.28
	DP-TQ (1.66mg/kg)	1.92 (1.1-3.3)	0.0073	0.021	17.34 (4.25/30.43)	0.013	0.24
Day 21	DP	1.63 (1.2-2.4)	0.26	*ref*	11.42 (-2.23/25.06)	0.096	*ref*
	DP-TQ (0.42mg/kg)	1.64 (0.6-3.1)	0.42	0.80	5.77 (-7.71/19.24)	0.38	0.54
	DP-TQ (0.83mg/kg)	1.64 (1-2.2)	0.017	0.65	16.3 (4.89/27.71)	0.0077	0.57
	DP-TQ (1.66mg/kg)	1.88 (1.3-2.9)	0.0065	0.30	11.5 (3.76/19.24)	0.0066	0.99
Day 28	DP	1.57 (1.1-2.1)	0.65	*ref*	5.76 (-6.14/17.65)	0.32	*ref*
	DP-TQ (0.42mg/kg)	1.57 (1-3)	0.46	0.91	9.46 (-5.05/23.98)	0.19	0.68
	DP-TQ (0.83mg/kg)	1.57 (1.1-1.9)	0.11	0.74	12.43 (1.16/23.71)	0.032	0.40
	DP-TQ (1.66mg/kg)	1.84 (1.3-3)	0.11	0.039	15.36 (-2.7/33.42)	0.091	0.35

Methaemoglobin concentration (Met Hb %) and percent change (relative to baseline) were compared within treatment arms (p-value¥) using paired t-tests (with day 0 as reference for percent change) and between treatment arms (p-value†) using linear regression (for Met Hb %, adjusted for baseline Met Hb %) or two-way t-tests (for percent reduction).

Supplementary figure 3. Haemoglobin and Methaemoglobin

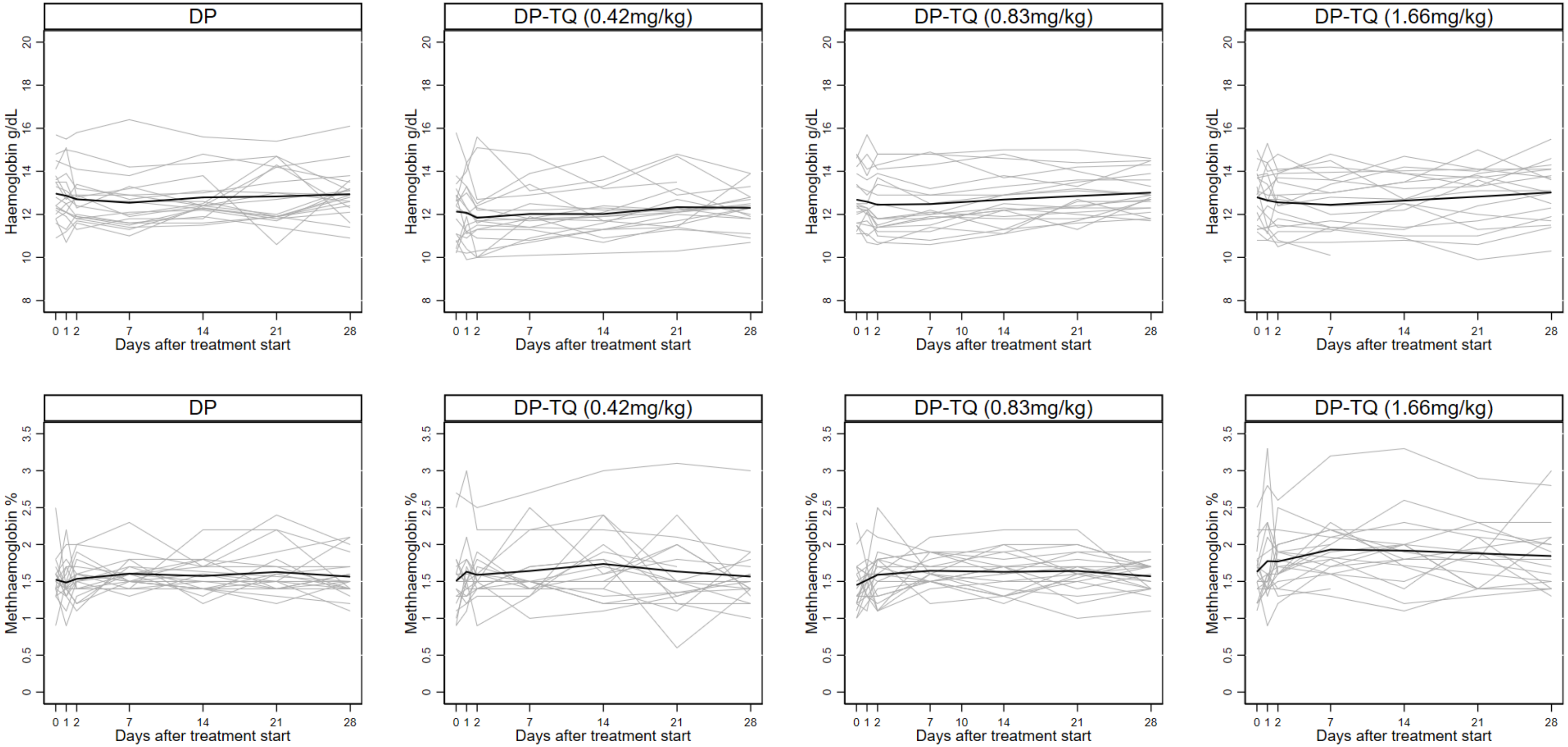

Absolute haemoglobin density is given in grams per dL (y axis, from 8-20g/dL) and is indicated for each participant individually with grey lines. The single black line shows the mean absolute haemoglobin density. P-values are presented in Supplementary table 6.

Supplementary table 8. Prevalence of adverse events

Description	All (n=80)	DP (n=20)	DP-TQ 0.42mg/kg (n=20)	DP-TQ 0.83mg/kg (n=20)	DP-TQ 1.66mg/kg (n=20)
**All**	69% (55/80)	60% (12/20)	75% (15/20)	65% (13/20)	75% (15/20)
P-value	*0.73**	-	*0.50***	*1.0***	*0.50***
**Mild AE**	65% (52/80)	60% (12/20)	70% (14/20)	60% (12/20)	70% (14/20)
P-value	*0.87**	-	*0.74***	*1.0***	*0.74***
**Moderate AE**	7.5% (6/80)	10% (2/20)	15% (3/20)	5% (1/20)	0% (0/20)
P-value	*0.50**	-	*1.0***	*1.0***	*0.49***
**Severe AE**	1.25% (1/80)	0% (0/20)	0% (0/20)	0% (0/20)	5% (1/20)
P-value	*1.0**	-	*nc*	*nc*	*1.0***
**All (related to treatment)**	40% (32/80)	30% (6/20)	50% (10/20)	35% (7/20)	45% (9/20)
P-value	*0.62**	-	*0.33***	*1.0***	*0.51***

P- values are from Fisher's exact tests for differences in proportion of individuals with an AE between all groups* or between TQ groups and the (DP) reference group **. Classification as 'related to treatment' was defined as probably, possibly or definitely related to treatment, as described in the methods. *nc* = not calculable.

Supplementary table 9. Frequency of all adverse events

	**Total**	**DP**	**DP-TQ 0·42mg/kg**	**DP-TQ 0·83mg/kg**	**DP-TQ 1·66mg/kg**
Abdominal pain	6^{4} (1)	5^{3} (1)	1^{1}	0	0
Allergic rhinitis	10	1	4	2	3
Diarrhea	2^{2}	0	1^{1}	0	1^{1}
Dizziness	4^{4}	2^{2}	1^{1}	0	1^{1}
Epigastralgia	1	0	0	0	1
Epistaxis	1	0	0	0	1
Fever	3^{1}(1^{1})	1	1^{1} (1^{1})	1^{1}	0
Gastroenteritis	2	0	1	1	0
Headaches	21^{14}(2^{2})	6^{2} (1^{1})	8^{5} (1^{1})	3^{3}	4^{4}
Hiccup	1	0	0	1	0
Nauseau	10^{10}	2^{2}	2^{2}	3^{3}	3^{3}
Pruritus	1^{1}	0	0	1^{1}	0
Rhinitis	7 (1)	2	2 (1)	1	2
Rhinobronchitis	5	2	0	0	3
Rhinorrhea	1	0	1	0	0
Acute otitis media	1	0	0	1	0
Salmonellosis	1	0	0	0	1
Pharyngitis	1 (1)	0	0	1 (1)	0
Tooth decay	2	0	1	1	0
Urinary tract infection	1 (**1**)	0	0	0	1 (**1**)
Vomiting	5^{3}	0	1^{1}	2	2^{2}
Wound	5	0	2	3	0
Elevated ALT	2^{2}	0	2^{2}	0	0
Elevated creatinine	1^{1} (1^{1})	0	1^{1} (1^{1})	0	0
ALL	94^{45}	21^{10}	29^{16}	21^{8}	23^{11}
MILD	*86^{41}*	*19^{9}*	*25^{13}*	*20^{8}*	*22^{11}*
MODERATE	*7^{4}*	*2^{1}*	*4^{3}*	*1*	*0*
SEVERE	*1*	*0*	*0*	*0*	*1*

55/80 participants experienced a total of 94 adverse events over the course of the trial; 86 categorised for severity by the study clinician (in accordance with the study protocol and data safety and monitoring charter) as 'mild', 7 as 'moderate', and 1 as 'severe'. No serious adverse events (SAE) occurred during the trial. The frequency of all AEs is given outside parentheses, with the frequency of moderate/severe AEs in parentheses (severe in bold and underlined). The frequency of AEs that were related to drug treatment (defined as probably, possibly or definitely related to treatment) is given in superscript. 45 of the 94 AEs were classified as possibly or probably related to the study drug; of these, 41/45 were mild, 4/45 moderate (headache, pharyngitis and elevated creatinine).

Supplementary table 10. Biochemistry

Day of follow-up	Treatment arm	Mean ALT IU/L (range)	p-value¥	p-value†	Mean AST IU/L (range)	p-value¥	p-value†	Mean creatine mg/dL (range)	p-value¥	p-value†
Day 0	*Overall*	19.84 (2-59)	-	-	23.64 (4-49)	-	-	0.67 (0.26-1.57)	-	-
	DP	21.46 (2-59)	*ref*	*ref*	24.39 (9-49)	*ref*	*ref*	0.68 (0.26-1.29)	*ref*	*ref*
	DP-TQ (0.42mg/kg)	20.52 (9-44)	*ref*	0.76	23.35 (6-41.3)	*ref*	0.74	0.66 (0.39-1.57)	*ref*	0.83
	DP-TQ (0.83mg/kg)	19.6 (9-46)	*ref*	0.59	24.9 (4-41)	*ref*	0.87	0.65 (0.4-1.13)	*ref*	0.71
	DP-TQ (1.66mg/kg)	17.8 (9-47)	*ref*	0.29	21.94 (9-48)	*ref*	0.43	0.69 (0.37-1.41)	*ref*	0.91
Day 2	DP	22.82 (9.5-40)	0.67	*ref*	26.1 (15-41)	0.57	*ref*	0.7 (0.33-1.07)	0.75	*ref*
	DP-TQ (0.42mg/kg)	28.54 (8-93.2)	0.12	0.13	30.49 (13-46)	0.0072	0.099	0.72 (0.39-1.38)	0.43	0.60
	DP-TQ (0.83mg/kg)	22.85 (9.1-46.4)	0.092	0.82	28.07 (12-48)	0.27	0.50	0.74 (0.35-1.24)	0.039	0.38
	DP-TQ (1.66mg/kg)	19.4 (4-51)	0.35	0.67	22.17 (4-38)	0.92	0.22	0.78 (0.46-1.38)	0.020	0.22
Day 7	DP	19.88 (11-29)	0.61	*ref*	25.83 (10-52)	0.67	*ref*	0.73 (0.45-1.2)	0.16	*ref*
	DP-TQ (0.42mg/kg)	29.26 (11-86.1)	0.059	0.0095	28.95 (6-59.3)	0.11	0.34	0.69 (0.5-1.12)	0.60	0.51
	DP-TQ (0.83mg/kg)	19.25 (7-47.6)	0.89	0.99	26.95 (7-45)	0.53	0.75	0.69 (0.35-1.06)	0.31	0.55
	DP-TQ (1.66mg/kg)	18.6 (7-35.3)	0.75	0.95	26.89 (17-43)	0.023	0.70	0.72 (0.34-1.21)	0.33	0.75
Day 14	DP	21.44 (5-37)	0.90	*ref*	23.25 (12-37)	0.76	*ref*	0.63 (0.41-0.93)	0.35	*ref*
	DP-TQ (0.42mg/kg)	18.55 (10-32)	0.41	0.35	22.89 (6-48)	0.99	0.98	0.59 (0.24-1.08)	0.42	0.58
	DP-TQ (0.83mg/kg)	22 (10-42.8)	0.38	0.73	27.31 (11-54)	0.44	0.17	0.63 (0.36-1.12)	0.49	0.91
	DP-TQ (1.66mg/kg)	17.38 (7-43.9)	0.63	0.24	23.74 (12-40.2)	0.48	0.75	0.73 (0.28-1.21)	0.73	0.18

Alanine aminotransferase (ALT), Aspartate aminotransferase (AST) and creatine were compared within treatment arms (p-value¥) using paired t-tests (with day 0 as reference) and between treatment arms (p-value†) using linear regression (adjusted for baseline levels). IU = international units.

References

1. Meerstein-Kessel L, Andolina C, Carrio E, Mahamar A, Sawa P, Diawara H, et al. A multiplex assay for the sensitive detection and quantification of male and female Plasmodium falciparum gametocytes. Malaria Journal. 2018;17(1):441.
2. Dicko A, Brown JM, Diawara H, Baber I, Mahamar A, Soumare HM, et al. Primaquine to reduce transmission of Plasmodium falciparum malaria in Mali: a single-blind, dose-ranging, adaptive randomised phase 2 trial. Lancet Infect Dis. 2016.
3. Dicko A, Roh ME, Diawara H, Mahamar A, Soumare HM, Lanke K, et al. Efficacy and safety of primaquine and methylene blue for prevention of Plasmodium falciparum transmission in Mali: a phase 2, single-blind, randomised controlled trial. Lancet Infect Dis. 2018;18(6):627-39.
4. Bousema T, Okell L, Shekalaghe S, Griffin J, Omar S, Sawa P, et al. Revisiting the circulation time of *Plasmodium falciparum* gametocytes: molecular detection methods to estimate the duration of gametocyte carriage and the effect of gametocytocidal drugs. Malaria Journal. 2010;9(1):136.
5. Méndez F, Muñoz A, Plowe CV. Use of area under the curve to characterize transmission potential after antimalarial treatment. The American Journal of Tropical Medicine and Hygiene. 2006;75(4):640-4.
